# Computational Reconstruction of NFκB Pathway Interaction Mechanisms during Prostate Cancer

**DOI:** 10.1371/journal.pcbi.1004820

**Published:** 2016-04-14

**Authors:** Daniela Börnigen, Svitlana Tyekucheva, Xiaodong Wang, Jennifer R. Rider, Gwo-Shu Lee, Lorelei A. Mucci, Christopher Sweeney, Curtis Huttenhower

**Affiliations:** 1 Department of Biostatistics, Harvard T.H. Chan School of Public Health, Harvard University, Boston, Massachusetts, United States of America; 2 The Broad Institute of MIT and Harvard, Cambridge, Massachusetts, United States of America; 3 Department of Biostatistics and Computational Biology, Dana-Farber Cancer Institute, Boston, Massachusetts, United States of America; 4 Department of Medical Oncology, Dana-Farber Cancer Institute, Harvard Medical School, Boston, Massachusetts, United States of America; 5 Department of Epidemiology, Harvard T.H. Chan School of Public Health, Harvard University, Boston, Massachusetts, United States of America; National Center for Biotechnology Information (NCBI), UNITED STATES

## Abstract

Molecular research in cancer is one of the largest areas of bioinformatic investigation, but it remains a challenge to understand biomolecular mechanisms in cancer-related pathways from high-throughput genomic data. This includes the Nuclear-factor-kappa-B (NFκB) pathway, which is central to the inflammatory response and cell proliferation in prostate cancer development and progression. Despite close scrutiny and a deep understanding of many of its members’ biomolecular activities, the current list of pathway members and a systems-level understanding of their interactions remains incomplete. Here, we provide the first steps toward computational reconstruction of interaction mechanisms of the NFκB pathway in prostate cancer. We identified novel roles for ATF3, CXCL2, DUSP5, JUNB, NEDD9, SELE, TRIB1, and ZFP36 in this pathway, in addition to new mechanistic interactions between these genes and 10 known NFκB pathway members. A newly predicted interaction between NEDD9 and ZFP36 in particular was validated by co-immunoprecipitation, as was NEDD9's potential biological role in prostate cancer cell growth regulation. We combined 651 gene expression datasets with 1.4M gene product interactions to predict the inclusion of 40 additional genes in the pathway. Molecular mechanisms of interaction among pathway members were inferred using recent advances in Bayesian data integration to simultaneously provide information specific to biological contexts and individual biomolecular activities, resulting in a total of 112 interactions in the fully reconstructed NFκB pathway: 13 (11%) previously known, 29 (26%) supported by existing literature, and 70 (63%) novel. This method is generalizable to other tissue types, cancers, and organisms, and this new information about the NFκB pathway will allow us to further understand prostate cancer and to develop more effective prevention and treatment strategies.

## Author Summary

In molecular research in cancer it remains challenging to uncover biomolecular mechanisms in cancer-related pathways from high-throughput genomic data, including the *Nuclear-factor-kappa-B* (NFκB) pathway. Despite close scrutiny and a deep understanding of many of the NFκB pathway members’ biomolecular activities, the current list of pathway members and a systems-level understanding of their interactions remains incomplete. In this study, we provide the first steps toward computational reconstruction of interaction mechanisms of the NFκB pathway in prostate cancer. We identified novel roles for 8 genes in this pathway and new mechanistic interactions between these genes and 10 known pathway members. We combined 651 gene expression datasets with 1.4M interactions to predict the inclusion of 40 additional genes in the pathway. Molecular mechanisms of interaction were inferred using recent advances in Bayesian data integration to simultaneously provide information specific to biological contexts and individual biomolecular activities, resulting in 112 interactions in the fully reconstructed NFκB pathway. This method is generalizable, and this new information about the NFκB pathway will allow us to further understand prostate cancer.

## Introduction

Proteins in the nuclear-factor-kappa-B (NFκB) complex belong to a family of transcription factors (NFκB1/p105, NFκB2/p100, RELA/p65, RELB, REL/c-REL) that regulate expression of genes involved in immune and inflammatory responses, cell growth, differentiation, and apoptosis. While these proteins are highly pleiotropic, their activation is context-specific [1]. The activation of NFκB protects against infection and stress, which is regulated by inhibitors of NFκB (IκB) proteins that keep NFκB inactive by binding to its protein complex, resulting in the phosphorylation of the IκBs by the IκB kinase (IKK) complex. Previous reports have shown NFκB to play an essential role in cancer by regulating the expression of genes involved in cell growth and proliferation, apoptosis, angiogenesis, and metastasis [2–5]. While the biomolecular activities and activation of the NFκB proteins have been studied previously [6,7], the NFκB pathway still remains incomplete.

Prostate cancer cells in particular have been reported to have constitutive NFκB activity due to increased activity of the IκB kinase complex, which can lead to cell growth and proliferation, while apoptosis is inhibited in prostate cancer cells [3,7–12]. Genome-wide methods, such as GWAS and expression studies, have linked a variety of NFκB-associated pathways to prostate cancer progression, including inflammatory processes (CXCL12, IL4, IL6, IL6ST, PTGS2, STAT3, and TNF) [13], cellular differentiation (LEPR, CRY1, RNASEL, IL4, and ARVCF) [14], and cell cycle regulation (FoxM1, SPP1) [15]. Within NFκB itself, p100 and p105 can mediate interaction with NFκB subunits that can also function as IκB proteins, and stimuli including cytokines, TLR signaling, and cellular stress can all activate or contribute to misregulation of the pathway [7]. Along with other inflammatory genes, signaling between NFκB and its regulators during inflammation [6,16–18] and cancer [7,19] has been the subject of close study, but neither the full repertoire of molecular players nor their mechanisms of interaction have been fully specified.

It is now possible to predict detailed, mechanistic interactions and pathway components using large-scale computational data integration [20,21]. This entails, for example, combining physical interaction and gene expression data with combinatorial and integrative approaches [22,23]. These methods have been previously used to predict a molecular signature of indolent prostate cancer [23] and biomarkers of metastatic breast cancer [22]. However, these efforts failed to take advantage of high-throughput experimental results from biological databases, which represent substantial resources for translational and bioinformatic research in clinical biomarker discovery and computational inference of biomolecular mechanism.

**Fig 1:**
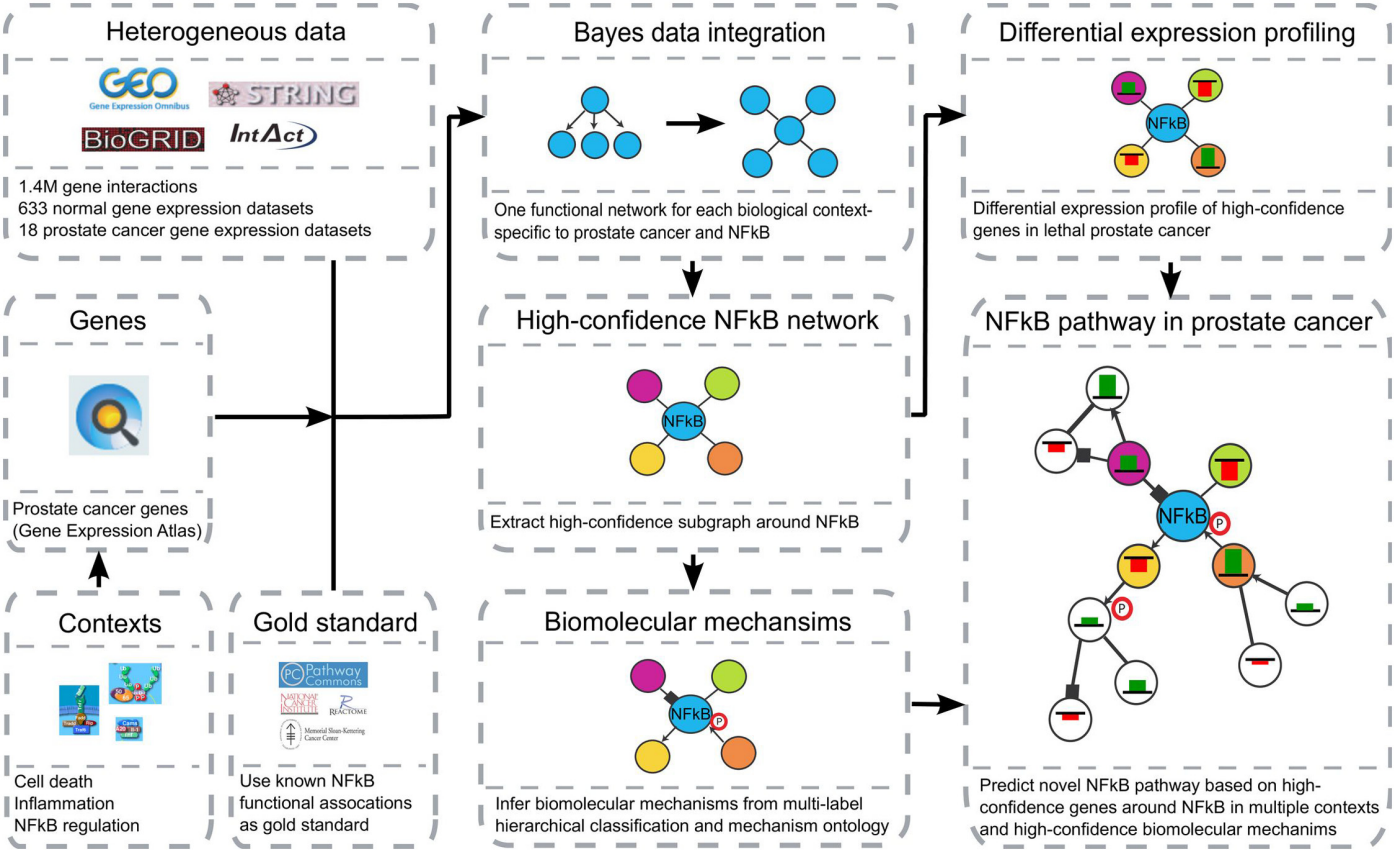
Computational recovery of mechanistic pathway components specific to the NFκB pathway as perturbed in prostate cancer. First, we integrated 18 prostate cancer-specific expression datasets and 633 non-disease datasets from expression (GEO), as well as 1.4M physical interactions (IntAct, BioGrid) and functional associations (Prosite, Domine, STRING), using prior knowledge from PathwayCommons which was refined by 9 biological processes as defined in Gene Ontology and by genes that were up- or downregulated in prostate cancer (Gene Expression Atlas). We trained naïve Bayesian classifiers and inferred context-specific functional networks related to prostate cancer and the NFκB pathway. Additionally, we applied an integrated method for concurrently predicting multiple protein interaction types [24] to assign a biomolecular mechanism to each functionally related gene pair in the final network. Here, we first trained individual Bayesian classifiers for each interaction type, followed by constructing a Bayesian network based on the ontology structure and fixed conditional parameters to constrain the hierarchical semantics of the ontology. Next, we extracted high-confidence subgraphs around NFκB for each context and identified those that are highly functionally related to NFκB in multiple contexts and determined their gene expression levels in an inhouse prostate cancer specific gene expression datasets from the Physicians^’^ Health Study (PHS) Prostatectomy Confirmation Cohort. Genes that were highly differentially expressed in this dataset (Table 1) were used to extract high-confidence subnetworks for each interaction mechanism, which were then combined as a novel NFκB pathway.

**Fig 2:**
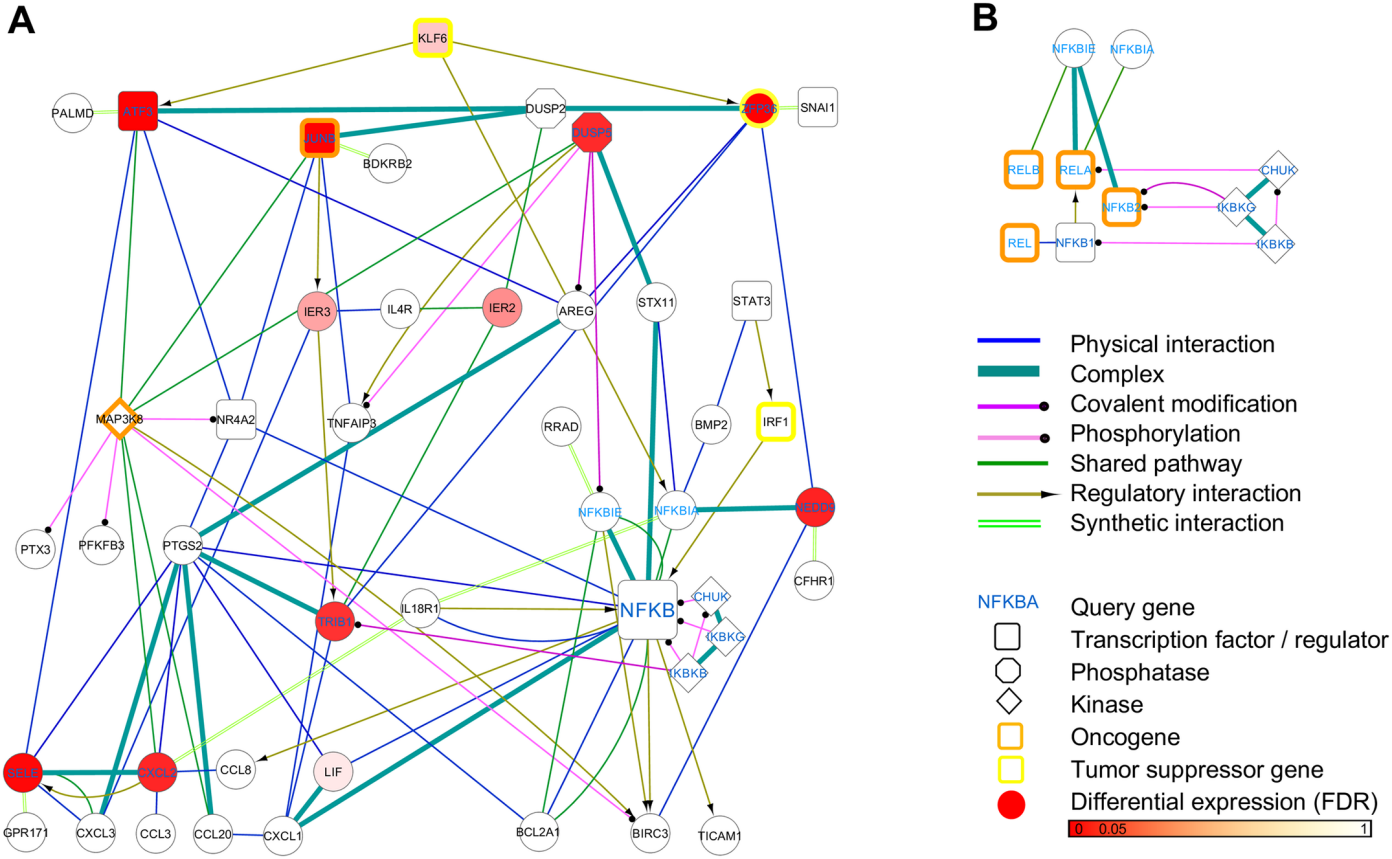
A novel predicted NFκB pathway specific to prostate cancer. (A) A pathway of putative molecular activities surrounding NFκB as predicted by our computational framework (Fig 1): the pathway results from a genome-wide functional interaction specific to the NFκB pathway in human prostate cancer and contains 50 genes connected by 112 mechanism-specific interactions. To generate this novel NFκB pathway, we extracted a high-confidence subnetwork from the genome-wide functional interaction network (see Methods) around 18 query genes in total (blue type), including five NFκB complex genes (NFκB1, NFκB2, REL, RELA, RELB), five NFκB specific inhibitors (NFκBIA, NFκBIE, IκBKB, IκBKG, CHUK), and eight genes found to be differentially expressed between lethal and indolent prostate cancer (Table 1). (B) We recovered all known molecular interaction mechanisms between NFκB complex members and their inhibitors (S17 Table).

In this study we address this challenge and provide the first steps toward computational recovery of mechanistic pathway components specific to the NFκB pathway as perturbed in prostate cancer (Fig 1). This was done by taking advantage of high-throughput experimental results from heterogeneous databases and training a model for specific biological contexts and specific to the NFκB pathway in prostate cancer. Here, we leveraged recent advances in Bayesian data integration [24] to simultaneously provide information specific to biological contexts and individual biomolecular mechanisms and applied this method to predict a novel NFκB pathway during its activity in cell death, inflammation, adhesion and differentiation as perturbed in prostate cancer. We integrated 651 gene expression datasets and 1.4M gene interactions in a context-specific manner using prior knowledge from known NFκB pathways. Focusing on genes differentially expressed in lethal prostate cancer versus indolent, we extracted a high-confidence pathway around such genes which are highly functionally related with the NFκB complex to predict a novel NFκB pathway specific to prostate cancer (Fig 2). Our predicted NFκB pathway suggested 8 novel genes which were found to be highly down-regulated in lethal prostate cancer and highly functionally related to NFκB, namely ATF3, CXCL2, DUSP5, JUNB, NEDD9, SELE, TRIB1, and ZFP36 (Table 1). Notable genes in the predicted pathway included ATF3, JUNB, KLF6, NR4A2, ZFP36, DUSP5 and NEDD9, as well as STAT3 and IRF1 as novel upstream regulators, and SELE, CXCL1 and CXCL2 as novel downstream targets of NFκB in prostate cancer. Connected by 112 predicted mechanistic interactions [13 (11%) previously known, 29 (26%) supported by existing literature, and 70 (63%) novel predictions (S17 Table)], these genes represent a promising and novel NFκB pathway as disturbed in human prostate cancer.

**Table 1:** List of genes significantly upregulated in lethal prostate cancer and highly confidently associcated with NFκB in multiple biological contexts. We integrated 860 total datasets (651 gene expression datasets and 225 gene interaction networks) using a Bayesian framework in different biological contexts (including cell death, cell differentiation, cell cycle, cell proliferation, cell migration, and NFκB regulation; S3 Table). From such context-specific networks, we extracted the subnetworks of genes most confidently associated with NFκB (S5 Table), which were subsequently analyzed in a differential expression study for significant (FDR corrected *p<0*.*05)* changes between lethal and indolent prostate cancer (see Methods); this resulted in eight total genes.

Gene	logFC	FDR	Biological contexts
ATF3	-0,96	0	Regulation of cell cycle, Cytokine metabolic process
CXCL2	-0,81	0,01	Vasculature development, Cytokine metabolic process
DUSP5	-0,76	0,02	Positive regulation of NFκB transcription factor activity, Cytokine metabolic process
JUNB	-0,81	0	Regulation of cell cycle, Positive regulation of NFκB transcription factor activity, Regulation of cell motion
NEDD9	-0,48	0,01	Vasculature development, Cell migration, Cytokine metabolic process
SELE	-0,83	0	Cell death, Regulation of cell proliferation
TRIB1	-0,35	0,02	Vasculature development, Cell migration, Regulation of cell motion, Cytokine metabolic process
ZFP36	-1,07	0	Regulation of cell cycle, Regulation of cell motion, Cytokine metabolic process

## Results

**Fig 3:**
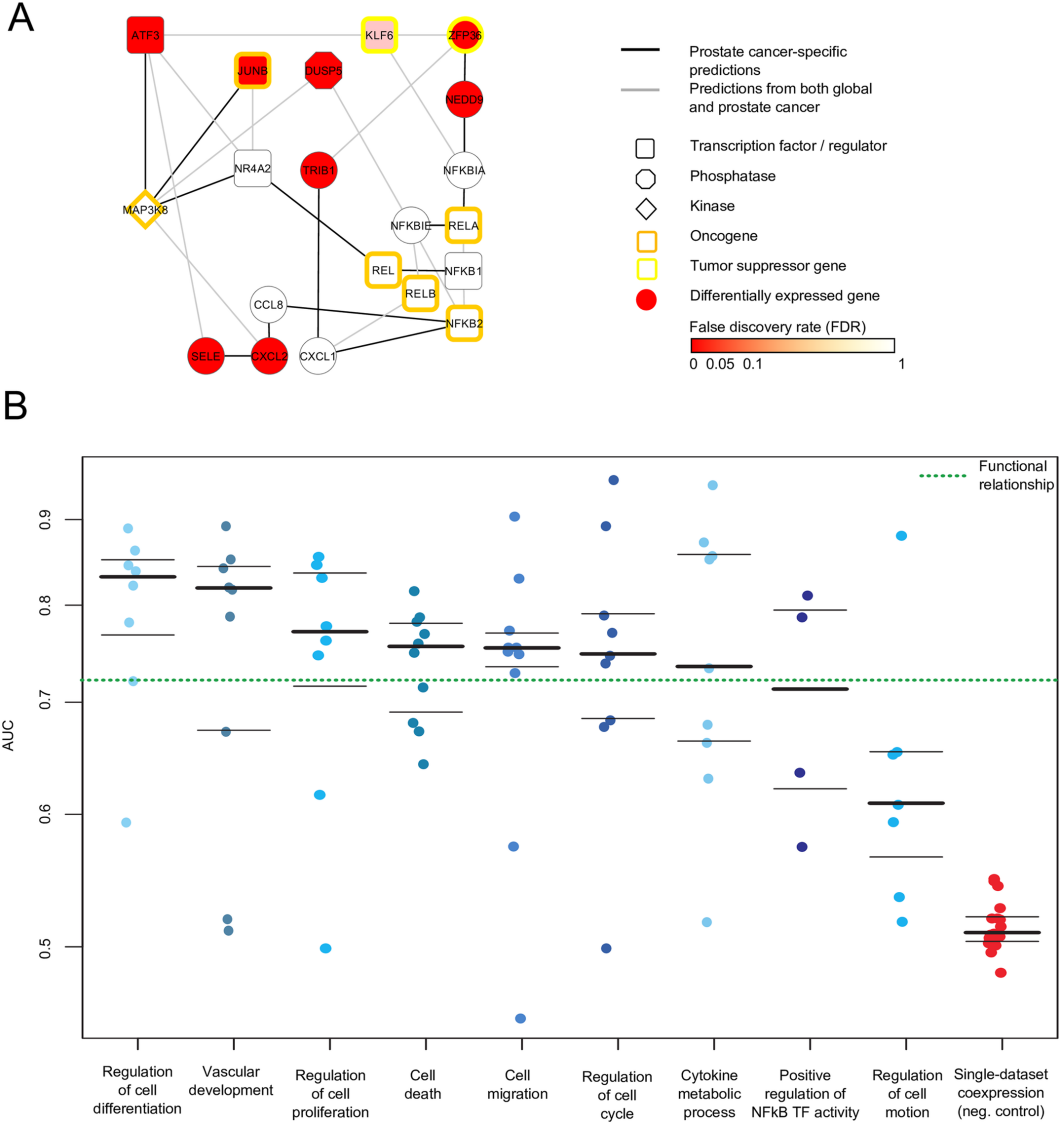
Context-specific functional networks for prostate cancer and the NFκB pathway. (A) Comparison between prostate cancer-specific and global (non-context-specific) predicted functional associations of 20 genes from our predicted NFκB pathway (Fig 2), including five NFκB genes (NFκB1, NFκB2, REL, RELA, RELB), two NFκB inhibitors (NFκBIA, NFκBIE), eight differentially expressed genes in lethal versus indolent prostate cancer (Table 1), and five additional connecting genes from our predicted NFκB pathway (MAP3K8, NR4A2, CCL8, CXCL1, KFL6) (Fig 2). We predicted 14 high-confidence functional associations between these genes that were exclusively context-specific to prostate cancer (black lines), along with 15 additional high-confidence (but not prostate cancer-specific) functional associations (grey lines). (B) Performance (AUC values) of the nine trained context-specific functional networks after performing a 10-fold gene-holdout-based cross-validation of each of the context-specific networks compared to a non-context-specific global functional network and, as a negative control, co-expression networks from 18 curated prostate cancer specific single expression data sets (S1 Table) revealed that the context specific networks are generally more accurate than the global functional network, while all trained networks outperform the negative control.

In this study we suggest a new mechanistic NFκB pathway in human prostate cancer (Fig 1). This pathway was derived from a functional relationship network predicted using regularized Bayesian integration [24] of high-throughput genomic data from 651 gene expression data sets and 1.4M gene interactions. These data simultaneously provided information on mechanisms of interaction (see Methods) of NFκB during its activity in cell death, inflammation, adhesion and differentiation. To predict a novel NFκB pathway as perturbed in prostate cancer, we focused on genes down-regulated in lethal prostate cancer (Fig 3A, Table 1) which were highly functionally related with NFκB in the integrated network (Fig 2). In this novel NFκB pathway we identified novel roles for ATF3, CXCL2, DUSP5, JUNB, NEDD9, SELE, TRIB1, and ZFP36 in this pathway, and predicted novel upstream regulators (ATF3, JUNB, KLF6, NR4A2, ZFP36, DUSP5 NEDD9, STAT3, and IRF1) and downstream targets (SELE, CXCL1 and CXCL2) of NFκB in prostate cancer, along with 70 (out of 112) novel mechanistic interactions (S17 Table).

### Extending the NFκB pathway in prostate cancer

The predicted NFκB pathway specific to prostate cancer consisted of 50 genes connected by 112 biomolecular mechanisms (Fig 2): of these mechanisms, 13 (11%) were previously known, 29 (26%) were supported by existing literature, and 70 (63%) were novel (S17 Table). In this pathway we show 10 known NFκB pathway genes (NFκB1, NFκB2, REL, RELA, RELB, IκB-α, IκB-ε, IKK-α, IKK-β, and IKK-γ; S16 Table), along with 8 novel genes that we found to be significantly down-regulated in lethal versus indolent prostate cancer in publically available databases [25,26] and that were highly functionally associated with NFκB in multiple biological contexts (Tables 1 and S12). Additionally, we recovered genes that previously have been reported to be associated with NFκB or prostate cancer (TNF-α/TNFAIP3, STAT3, MAP3K8, NR4A2/NR3C4, BCL2, and IL18; S16 and S17 Tables), as well predicted novel upstream regulator genes (ATF3, JUNB, KLF6, NR4A2, ZFP36, DUSP5 and NEDD9, and IRF1) and downstream target genes of NFκB (SELE, CXCL1 and CXCL2; S16 Table) which may be involved in the NFκB pathway for development and progression of lethal prostate cancer as detailed below.

As a first step, we tested whether the genes newly predicted to this pathway were enriched for pathways, diseases, or biological processes from the Gene Ontology, KEGG, or the Pathway Interaction Database associated with tumorigenesis or prostate cancer [27] (S13A Table). Interestingly, this analysis revealed that many of the highly enriched biological processes and molecular functions from Gene Ontology were related to inflammation and innate immunity (S13B Table): processes in which chemokines and cytokines play an important role [28], of which CCL20, CCL3, CCL8, CXCL1, CXCL2, and CXCL3 occurred in our pathway (Fig 2). Additionally, we observed strong functional enrichments in the extracellular space, a major component in cancer development and progression [29]) for AREG [30,31], SELE [32], LIF [33], and several chemokines and cytokines [34–38], as well as high disease enrichment (NCI Cancer Gene Index) in 47 different cancer associations from all major tissues (S13B Table), indicating that these genes are not only involved in prostate cancer, but also in a variety of other cancer types.

### Recovery of known NFκB and prostate cancer related genes and their interaction mechanisms in the predicted pathway

The NFκB complex consists of 5 proteins [NFκB1 (p105), NFκB2 (p100), RELA (p65), RELB and REL (c-Rel)] and, upon activation, provides a powerful defense mechanism against infection and stress; regulation of the complex in managed in part by families of NFκB inhibitor genes (IκB) and kinases (IKK) [4,7]. Here, our predictions suggested that the IκB genes [NFκBIA (IκB-α) and NFκBIE (IκB-ε)] directly bind to NFκB and regulate (inhibit) NFκB upstream to maintain an inactive state, while IKK kinases (CHUK/IKK-α, IκBKB/IKK-β, and IκBKG/IKK-γ) phosphorylate NFκB for downstream activation, which is in line with previous reports [7,39]. We not only recovered these NFκB complex genes, their inhibitors, and their correct biomolecular mechanisms, but also identified 8 additional genes as significantly down-regulated in lethal prostate cancer and highly functionally associated with NFκB in multiple biological contexts (Table 1). 40 additional novel genes were suggested to constitute a novel NFκB pathway in prostate cancer. Within this pathway, we predicted 112 interactions’ biomolecular mechanisms, out of which we could verify 29 (26%) based on previous studies, while 70 (63%) were novel. Along these 70 novel interactions, 18 gene pairs were reported in other literature as co-regulated without an explicit mechanism of interaction (S17 Table).

In particular, our results predicted BCL2 and several inflammatory chemokines to be novel downstream targets of NFκB, including the anti-apoptotic protein BCL2A1, the chemokine (C-X-C motif) ligand 1 (CXCL1), and (C-C motif) ligand 8 (CCL8). This conclusion is based on a predicted direct binding and downstream regulation of BCL2A1 by the NFκB complex (in particular the REL, RELB, and NFκBIE subunits), a predicted downstream regulation of CCL8 by NFκB2, and a direct binding of CXCL1 with NFκB2 and RELB (Fig 2). This is in line with previous findings that BCL2 expression is dependent upon REL and RELA [40] to promote resistance to programmed cell death and important pro-survival functions [7,41], while BCL2L1 (BCL-XL), another anti-apoptotic protein, was observed to be upregulated by NFκB as a critical link between inflammation and cancer [4] and tumor progression [41]. In addition, previous studies showed that IKK-NFκB signaling pathways may lead to downstream upregulation in expression of certain tumor-promoting cytokines and survival genes, including BCL2 and inflammatory chemokines [4,42] (as predicted in this study). We were able to further confirm reports that the NEMO-dependent NFκB pathway regulates the expression of many proinflammatory genes, including CCL8, CXCL2, CCL2, SELE, and several interleukins [43]. Specifically, these reports are complementary with our predictions that inflammatory chemokines directly interact with each other (e.g. CXCL1 and CCL20, CCL8 and CXCL2, CXCL2 and CCL3), while CXCL2 was predicted to directly bind or regulate SELE, and IL18R1 was predicted to co-regulate NFκB jointly with CXCL2 in a feedback loop (Fig 2). In this predicted feedback loop we found that IL18R1, an interleukin receptor binding to the IL18 gene, regulates REL and RELB and directly binds to NFκB1, which can be supported by previous findings [44].

In addition to downstream targets of NFκB, we also recovered important upstream regulators for cancer development and progression, including STAT3, MAP3K8, and TNF. Our predictions suggested that STAT3 (signal transducer and activator of transcription) and the bone morphogenetic protein BMP2 concordantly influence NFκB in prostate cancer by predicted direct interaction, which complements previous studies showing that BMP2 induces apoptosis with modulation of STAT3 [43]. Additionally, STAT3 was predicted to regulate the transcription factor and tumor suppressor gene IRF1 (interferon regulatory factor 1), as previously confirmed [45], which was predicted to regulate NFκB (Fig 2). Based on this prediction we suggest that STAT3, BMP2, and IRF1 concordantly regulate NFκB activation upstream in prostate cancer, as it was shown that mechanisms that underline the oncogenic functions of NFκB are likely to require additional transcription factors such as STAT3, which can function cooperatively with NFκB, and are likely to help to drive NFκB-dependent tumorigenesis [7,46].

The oncogene MAP3K8 was correctly predicted as an upstream activator of NFκB and activator of both the MAP kinase and JNK kinase pathways, which leads to an activation of downstream genes such as c-Jun, and JUNB, an AP1 transcription factor and oncogene [47,48]. This is based on a predicted phosphorylation of the nuclear receptor NR4A2 gene, a family member of AR (NR3C4), and regulation of JUNB and DUSP5, all predicted genes to act as novel upstream regulators of NFκB (see above), suggesting that MAP3K8 would be another important upstream regulator of NFκB in prostate cancer.

Another gene that was predicted to directly interact with the JNK kinase pathway, in particular JUNB [49], was the tumor necrosis factor alpha induced protein TNFAIP3 (A20), a known inhibitor of NFκB activation [50]. In this case, we did not observe a direct regulation of NFκB by TNFAIP3, but rather an indirect interaction, as we predicted that TNFAIP3 physically interacts with the chemokine CXCL1 [50–52], which is a predicted downstream target of NFκB (see above). Our prediction did not reveal a direct upstream nor downstream effect of TNFAIP3 on NFκB, which may be the result of its functional role in negative feedback loops [7].

### Genes newly predicted to act as NFκB regulators or target genes in prostate cancer

We predicted several genes to act in prostate cancer as novel upstream regulators of NFκB or novel downstream regulatory targets of NFκB. These included 8 genes differentially expressed in lethal prostate cancer and highly functionally related to NFκB (Table 1) along with additional promising candidates such as CXCL1, KLF6, and IRF1 (S16 Table). Among these genes, our predictions highlight ATF3, JUNB, KLF6, NR4A2, ZFP36, DUSP5, NEDD9, STAT3 and IRF1 as novel and promising upstream regulators of NFκB in prostate cancer, while SELE and the chemokines, including CXCL1 and CXCL2, act as novel downstream targets of NFκB in prostate cancer.

In particular, we predicted the nuclear receptor NR4A2 and the activating transcription factor 3 (ATF3) as novel upstream regulators of NFκB in prostate cancer. This was based on the observation that ATF3 was predicted to be highly functionally related to NFκB during regulation of cell cycle and cytokine metabolic process (Table 1) and to indirectly bind to NFκB via NR4A2 after phosphorylation by MAP3K8 (Fig 2); notably, previous findings only observed ATF3 as a co-repressor with NFκB in prostate cancer [53]. The tumor suppressor gene and transcription factor KLF6 (Kruppel-like factor 6) is another gene that we predicted to directly regulate not only ATF3 (as previously suggested [54]) but also NFκB inhibitor α, and which we therefore suggest as another important upstream regulator of the NFκB cascade in prostate cancer. Another gene predicted to act as an upstream inhibitor of NFκB via NR4A2 was the transcription factor and proto-oncogene JUNB (Fig 2), which appeared to be highly functionally related to NFκB in multiple biological contexts, including the positive regulation of NFκB transcription factor activity (Table 1). However, instead of a direct interaction with NFκB as observed in previous studies [55,56], we predicted an indirect inhibition of NFκB via NR4A2 as a mechanism in prostate cancer suppression. These novel predicted genes that regulate NFκB via NR4A2 suggest a key role of this nuclear receptor within the NFκB pathway. NR4A2 is a family member of AR (NR3C4), which is known to be activated downstream of the MAPK pathway in cancer [57] and directly interacts with NFκB (specifically the REL subunit), as correctly predicted for NR4A2 [58]. Along with these genes (ATF3, JUNB, and NR4A2) that we predicted to be regulated by the MAP kinase MAP3K8, a known oncogene involved in prostate cancer growth [48], we additionally suggest the dual specificity phosphatase 5 (DUSP5) as another upstream regulator of NFκB in prostate cancer: DUSP5 was not only predicted to be highly functionally related to NFκB in the positive regulation of NFκB transcription factor activity (Table 1), but also correctly predicted to be regulated by MAP3K8 (Fig 2) [59].

We correctly predicted and confirmed the NFκB inhibitors ε (NFκBIE) [60] and α (NFκBIA) [61,62] as upstream regulators of NFκB (see above) and also predicted NEDD9 (neural precursor cell expressed, developmentally down-regulated 9) as another upstream regulator of NFκB that acts by directly binding to an NFκB inhibitor, NFκBIA (Fig 2). Additionally, NEDD9 was predicted to interact directly with the zinc finger protein 36 homolog (ZFP36), a tumor suppressor gene that negatively regulates NFκB [63,64]. This is complementary with our predictions, suggesting that after being regulated by KLF6, ZFP36 directly binds to NEDD9 (Fig 2), thus acting as another novel upstream inhibitor of NFκB with a role in the amelioration of prostate cancer.

In addition to these novel upstream regulators of NFκB in prostate cancer, we also predicted new downstream targets, including several cytokines and a selectin. Our prediction of the chemokine (C-X-C motif) ligand 1 and 2 (CXCL1, CXCL2) as direct and indirect downstream targets of NFκB in prostate cancer can be supported by previous finding in different contexts [7,51,65,66]. The selectin E gene (SELE) was predicted to be downstream regulated by NFκB via such chemokines (CCL8, CXCL2, CXCL3) (Fig 2), while previous studies observed that it activates the PI3K/NFκB pathway in colon cancer [67]. However, SELE is found in cytokine-stimulated endothelial cells and is thought to be responsible for the accumulation of blood leukocytes at sites of inflammation [68], supporting our confident predicted relationship between SELE and cytokines in this pathway. As CXCL1 and CXCL2 were predicted as downstream targets of NFκB in prostate cancer, we suggest SELE as another important downstream target in this process. Additionally, one of these predicted downstream chemokines, CXCL1, was predicted to directly bind to the Human Tribbles homolog 1 (TRIB1), a gene that is reportedly involved in the regulation of NFκB and MAP kinases [69]. This report agrees with our prediction and suggests that TRIB1 could be posttranslationally modified by IKBκB, an NFκB inhibitor, providing an indirect effect of NFκB in prostate cancer.

### Functional data integration for pathway component predictions

We inferred NFκB pathway components in prostate cancer using information from 860 total datasets. 651 of these were gene expression studies, of which 18 were included specifically due to profiling prostate cancer tissues. We additionally incorporated 225 interaction networks (protein-protein, regulatory, and genetic interactions) together comprising 1.4M interactions. These data were unified into a predicted set of pathway-specific interactions using a Bayesian framework to model the probability of each dataset providing accurate results relevant to disease pathways in prostate cancer [70,71]. This procedure automatically down-weights noisy datasets and those not relevant in a particular context, ultimately providing a single model within which many different types of interaction mechanisms can be captured. One context-specific network [cell death, cell differentiation, cell cycle, cell proliferation, cell migration, and NFκB regulation (S3 Table)] was produced for each interaction mechanism in this study, using the independent subset of data in each case (see Methods). From such predicted functional relationship networks specific for interaction mechanisms, we extracted genes highly confidently related with a set of predefined query genes [NFκB, IκB, and 8 down-regulated genes (Tables 1 and S12)], which were integrated into one NFκB pathway as outlined in Fig 1 and illustrated in Fig 2 (see Methods for addition details).

### Context specificity and validation

To identify interactors within the NFκB pathway in each context, we extracted the subnetworks most confidently associated with the NFκB1 gene, i.e. its nearest neighbors, which resulted in 66 genes in total (S5 Table). Among these genes, most were highly confidently associated with NFκB in multiple contexts (S5 Table). For example, CCL20, a cytokine regulated by other inflammatory cytokines (e.g. TNF, INF, or IL-10) [72] was highly associated with NFκB in vasculature development, cell migration, positive regulation of NFκB TF activity, and regulation of cell motion, while the transcription factor and proto-oncogene JUNB showed strong association with NFκB in the context of cell cycle and cell motion regulation, as well as positive regulation of NFκB transcription factor activity, which is in line with previous findings [55,56].

Next, we analyzed which of these 66 genes showed a significant change in gene expression (at a significance level of 5% after FDR correction) between lethal and indolent prostate cancer (see Methods) [25,26], which resulted in a set of 8 genes: cyclic AMP-dependent transcription factor (ATF3), chemokine (C-X-C motif) ligand 2 (CXCL2), dual specificity protein phosphatase 5 (DUSP5), transcription factor jun-B (JUNB), enhancer of filamentation 1 (NEDD9), e-selectin (SELE), tribbles homolog 1 (TRIB1), and zinc finger protein 36 homolog (ZFP36; Table 1). All of these genes were down-regulated in patients who had disease that relapsed after a prostatectomy, which could be the result of negative feedback loops in lethal prostate cancer that turn off important cancer regulators, such as ZFP36, DUSP5, and ATF3 [7]. Surprisingly, none of the NFκB genes were significantly differentially expressed (NFκB1: FDR = 0.69, NFκB2: FDR = 0.19, REL: 0.60, RELB: FDR = 0.71, RELA: FDR = 0.91) (S7 Table), which could be a result of their constitutive activation, negative feedback loops, or the presence/absence of cancer regulator genes that determine whether it promotes cancer to develop metastatic disease [7].

The eight genes found to be significantly down-regulated in prostate cancer (Table 1) were further explored in a meta-analysis based on the Gene Expression Atlas (GXA) [73,74]. This database of meta-analysis is based on summary statistics over a curated subset of ArrayExpress Archive, servicing queries for condition-specific gene expression patterns as well as broader exploratory searches for biologically interesting genes/samples. Additionally, this meta-analysis revealed that a subset of the eight genes (ATF3, CXCL2, JUNB, and ZFP36) were significantly up-regulated in normal (non-disease) prostate tissue (S16 Table), further supporting their role as high-confidence regulators of NFκB in prostate cancer.

#### Validation

To quantitatively assess the expected performance of our predicted context-specific networks (Fig 3A), we performed a 10-fold gene-holdout-based cross-validation, yielding an average AUC of 0.75±0.07 (95% CI) across all contexts (Fig 3B, S14 Table). We also compared each of the context-specific networks to a non-context-specific global functional network and, as a negative control, co-expression networks from 18 curated prostate cancer-specific expression datasets (Fig 3B, S1 and S14 Tables). This analysis revealed a higher accuracy for the context-specific networks (average AUC = 0.75±0.07 across all contexts) than for the global functional network (AUC = 0.72±0.02), while all of these networks outperformed the negative control (AUC = 0.51±0.01). In particular, we observed the highest AUC values for the context-specific networks “Regulation of cell differentiation” (AUC = 0.83±0.06) and “Vascular development” (AUC = 0.82±0.09), while “Regulation of cell motion” (AUC = 0.61±0.08) showed the smallest AUC value among the context-specific networks (S14 Table), but still higher than the negative control (AUC = 0.51±0.01). We did not observe a correlation between the context-specific AUC values and the size of the context-specific gene sets used to refine the underlying gold standard for training the context-specific classifiers (see Methods and S3 Fig).

### Mechanism specificity and validation

**Fig 4:**
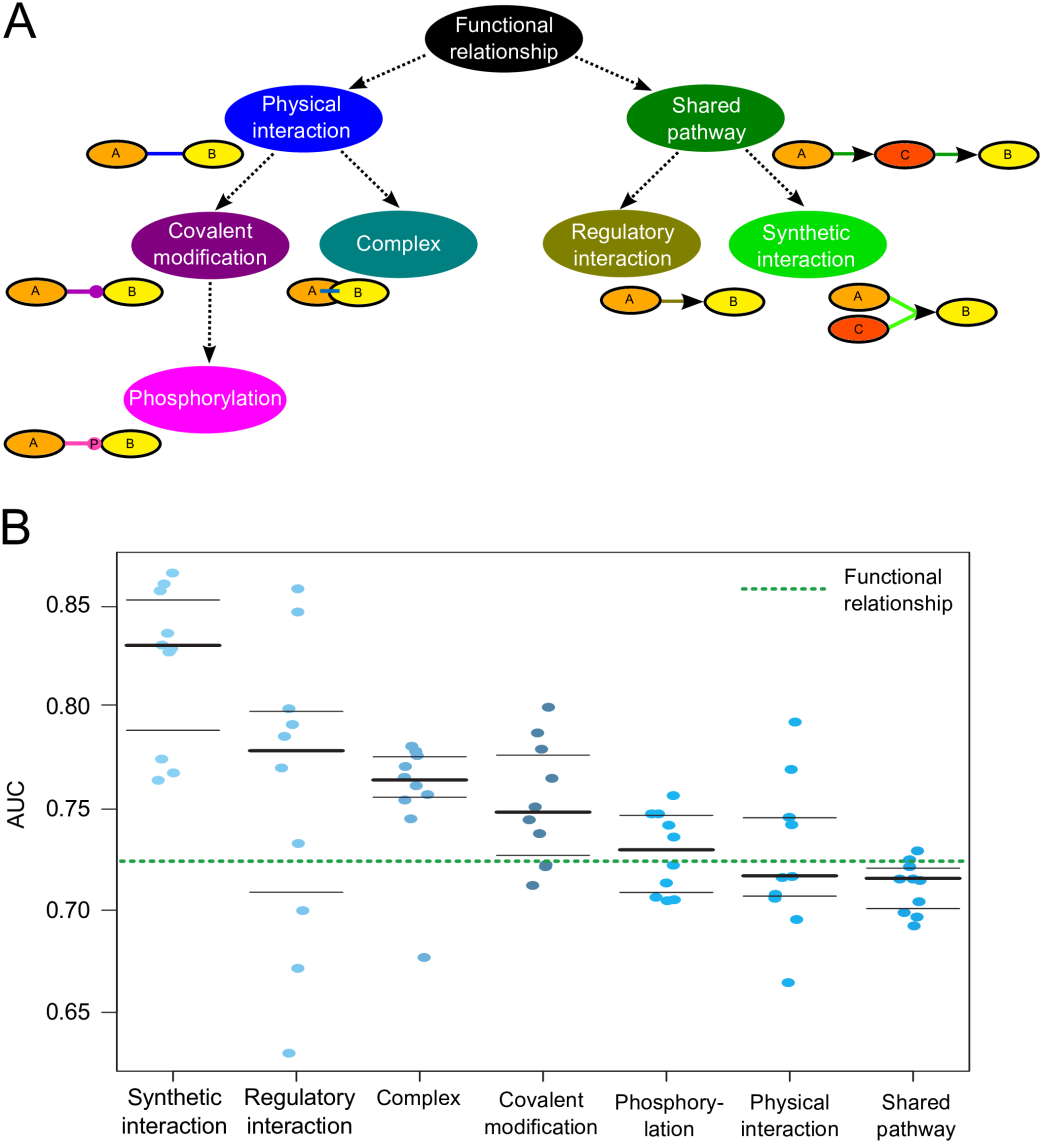
Mechanistic interaction networks specific to prostate cancer and the NFκB pathway. (A) We constructed an interaction ontology containing seven interaction types: a functional relationship can be a physical interaction (two genes directly bind to each other and interact physically), a complex (two genes form a protein binding complex), a covalent modification (a kinase posttranslationally modifies a substrate), a phosphorylation (a kinase adds a phosphate group to a substrate at a phosphorylation site), a shared pathway (two genes react in the same pathway which can be an indirect regulation), a regulatory interaction (a gene is activating or inhibiting another gene), or a synthetic interaction (two genes simultaneously regulate another gene, whereas the two genes individually would not regulate the third gene). (B) The performance (AUC values) of the seven trained mechanistic interaction networks after performing a 10-fold gene-holdout-based cross-validation of each of the networks revealed that the mechanistic interaction networks are generally more accurate than the global functional network.

As outlined above, we produced one context-specific network for each interaction mechanism based on our interaction ontology (Fig 4A) and using the independent subset of data in each case (see Methods). To take the ontology of interaction mechanisms into account, we applied a multi-labeled hierarchical classification formulation enabling us to infer one mechanism-specific network for each interaction type while keeping conserved and non-conserved gene pairs in child-parent relationships in the interaction ontology (see Methods). To identify interactors with NFκB in each interaction mechanism, we extracted the subnetworks most confidently associated with the NFκB1 gene from each mechanism-specific network, which we then integrated into one NFκB pathway (see Methods and Fig 2). The resulting pathway consisted of 50 genes in total [including all NFκB complex genes and its inhibitors, as well as the 8 significantly upregulated genes as derived above (Table 1)] connected by 112 non-redundant interactions from 7 different biomolecular mechanisms (Fig 4A).

#### Validation

To assess the performance of these predicted mechanism-specific networks, we performed a 10-fold gene-holdout-based cross-validation, yielding an average AUC of 0.75±0.02 (95% CI) across all mechanisms (Fig 4B, S14 Table). Comparing each individual interaction network to a global functional network (AUC = 0.72±0.02) (Fig 4B) revealed that 5 out of 7 mechanistic networks were performing equal to or better than the global functional network (AUC≥0.72), while two mechanism-specific networks (“Physical interaction” and “Shared Pathway”) did not perform significantly differently (both AUC = 0.71; Fig 4B, S14 Table). We identified the mechanisms “Synthetic interaction” (AUC = 0.83±0.03) and “Regulatory interaction” (AUC = 0.77±0.05) as the two best-performing mechanism-specific networks, while the two less-specific interaction types “Physical interaction” and “Shared Pathway” (both AUC = 0.71) were performing worse than the more specific interactions and the global interaction network (Fig 4A).

In line with a previous study [24], these results illustrate that the more specific interaction types within the interaction ontology generally perform better than the less specific interaction types, which is the positive result of the multi-labeled hierarchical classification formulation (see Methods). Additionally, in agreement with the predictions of the context-specific networks, we could not find a correlation between the interaction-specific AUC values and the size of the gold standard gene sets (S5 Fig).

#### Network analysis of mechanistic interactomes

As outlined above and detailed in Methods, we predicted individual mechanism-specific networks based on a multi-labeled hierarchical classification formulation [24,75]. This approach trains an individual Bayesian classifier for each interaction type based on high-throughput and heterogeneous genomic datasets and a corresponding mechanistic gold standard. As biological networks generally have a scale-free topology and follow a power-law distribution [76,77], we investigated these properties in our predicted mechanism-specific networks, revealing scale-free distributions for all interaction types (S1 Fig), which is also in line with previous findings [24]. Analyzing the hub genes within these mechanism-specific networks, the overlap of the top 5% of high-degree hubs between all interaction networks as hub genes differed minimally across biomolecular mechanisms, with high-degree hub genes overlapping to a large degree between ontology-related mechanism-specific networks such as physical interactions and complex, or covalent modification and phosphorylation. We observed much less overlap of hub genes between regulatory interactions and physical interactions (S2 Fig).

#### Estimating true and false positive predictions in a quality control analysis

To computationally estimate the true and false positive prediction rates of our approach, we compared our predictions with (1) a prostate cancer-specific co-expression analysis, (2) known mechanisms from the NFκB signaling pathway in BioCarta (BioCarta.com, BioCarta LLC), and (3) known mechanisms from literature. This revealed a high area under the curve (AUC) value of 0.83 after comparing our predictions with co-expression, along with true positives for 78.5% of all positive predictions with a precision rate of 66.1% for the interactions from BioCarta and PubMed, as detailed below.

First, we compared our predicted NFκB pathway with gene co-expression relationships retrieved from the TCGA dataset (see Methods). This enabled us to compare gene pairs that are co-expressed in lethal prostate cancer (and thus, functionally related on a transcriptional level) with our predictions that they are functionally associated in the context of NFκB in prostate cancer and relevant biological contexts. This analysis revealed a strong performance (AUC = 0.83, S6 Fig): 70% of predicted high-confidence interactions were found to be co-expressed in the TCGA dataset, while <10% of randomly chosen low-confidence predictions were co-expressed (S15 Table, S7 Fig).

Second, we compared our predictions with reported interactions from the NFκB signaling pathway in BioCarta and literature (see Methods). This comparison revealed a true positive rate (known edges in the NFκB signaling pathway in BioCarta or in the literature) of 81% and precision of 67% (S8 Table). Notably, pairs of proteins can interact by more than one mechanism, and multiple such mechanisms were predicted by our approach. For example, NFκB1 and RELA can form a protein complex [78], NFκB1 can regulate RelA acetylation through phosphorylation [79], and NFκB can be regulated through interaction of RELA with histone deacetylase (HDAC) corepressor proteins [80]. While, BioCarta reports only the protein complex between NFκB1 and RELA, we predicted all three mechanisms (complex, phosphorylation, and regulation) with high-confidence. Additionally, of 28 pairs of NFκB proteins not reported to interact in BioCarta (True Negatives; S9 Table), 19 were non-interacting in our predictions, while nine were predicted to have high-confidence interactions (False positives). Notably, one of our nine “false positive” predictions—i.e. that MAP2K1 phosphorylates FADD in prostate cancer—was also reported in an independent study [81]. This suggests that the gold standard negatives from BioCarta are incomplete and that our true negative rate serves as a conservative estimate.

Third, we compared 50 known NFκB interaction mechanisms from the literature (NCBI) with our predictions (see Methods; S10 and S11 Tables), revealing 80% predicted true positives (TPs) for known NFκB1 interactions and 72% predicted TPs for known TNF interactions with precision rates of 64%. For example, our method predicted that NFκB1 and the B-cell lymphoma 2 (BCL2) gene would interact within the same pathway with high-confidence (0.99; S10 Table), which could be established in a recent study [82]. As another example, we correctly predicted that Tumor necrosis factor-α (TNF-α) and transforming growth factor-β (TGF-β) were synthetically interacting in the same pathway (S11 Table), as a recent study revealed that TGF-β and TNF-α act in concert to activate apoptosis in cancer [83]. Additionally, our predictions revealed a high-confidence regulatory interaction between TNF and FOXP3, which is also in line with previous findings [84].

### Experimental validation of novel predicted mechanisms

**Fig 5:**
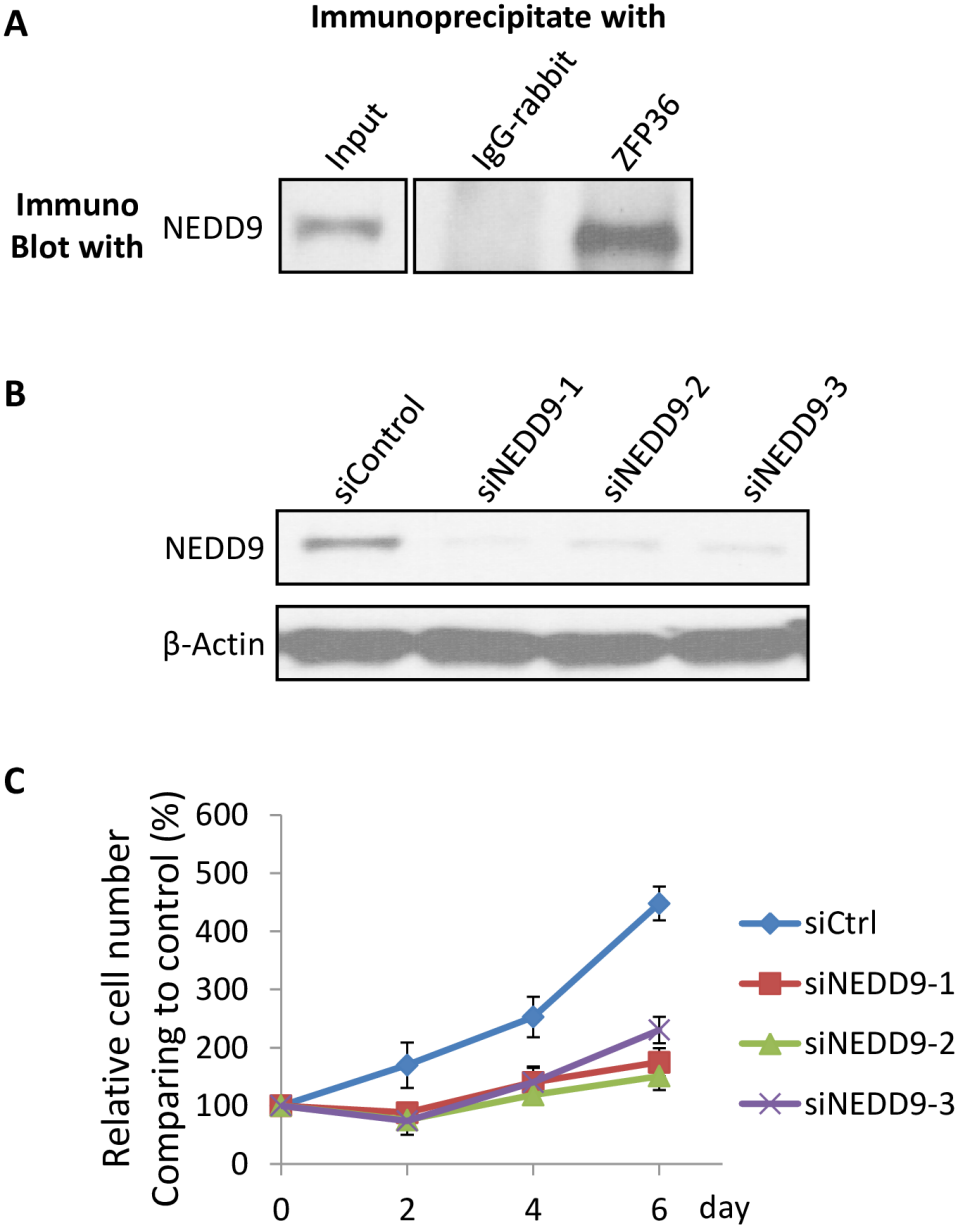
NEDD9 / ZFP36 co-immunoprecipitation supports predicted physical interaction and knockdown regulates cell proliferation in a prostate cancer line. (A) Anti-ZFP36 was used to co-immunoprecipitate ZFP36-NEDD9 complex, confirming the presence of NEDD9 by western blot; IgG-rabbit antibody was included as a negative control. (B) LAPC4 cells were transfected with NEDD9 and control siRNAs (see Methods), with knockdown efficiency verified by western blot. (C) Cell proliferation rate measured after NEDD9 knockdown by WST-1 optical density (see Methods). Depletion of NEDD9 transcript consistently downregulates proliferation rates and suggests a possible role in growth regulation.

To validate our computational reconstruction of novel interactions, we chose to assay the potential interaction between NEDD9 and ZFP36 by co-immunoprecipitation (based on the availability of antibodies and their relative expression levels in the cell model system; Fig 5). NEDD9 was confirmed by western blot during immunoprecipitation by anti-ZFP36 antibody (Fig 5A), supporting the association of NEDD9 with the protein complex identified by anti-ZFP36. NEDD9 was also suggested to play a role specifically in prostate cancer cell proliferation. After successful knockdown of NEDD9 by siRNA in the LAPC4 prostate cancer cell line (Fig 5B), proliferation was significantly inhibited relative to control (Fig 5C). These preliminary validation studies thus support our computational reconstructions and the predicted new roles and interactions of at least these two genes newly characterized in the NFκB pathway in prostate cancer.

## Discussion

In this study we provide the first steps toward computational recovery of mechanistic pathway components specific to the NFκB pathway as perturbed in prostate cancer. We used a Bayesian data integration model to simultaneously provide information specific to biological contexts and individual biomolecular mechanisms for predicting a novel NFκB pathway during its activity in prostate-related biological contexts, including cell death, inflammation, adhesion and differentiation. Our predicted NFκB pathway (Fig 2) revealed 8 genes highly down-regulated in lethal prostate cancer and highly functionally related to NFκB (Table 1), including novel upstream regulators (ATF3, JUNB, KLF6, NR4A2, ZFP36, DUSP5, NEDD9, STAT3 and IRF1) and novel downstream targets (SELE, CXCL1 and CXCL2) of NFκB in prostate cancer.

The identification of disease- and tissue-specific pathways remains a challenging problem—one which we addressed here in the context of a prostate cancer specific NFκB pathway. Historically, automated pathway reconstruction has required extensive expert knowledge and manual curation. Although there exist several pathway collections and databases (e.g. BioCarta, KEGG [85], Reactome [86], NCI Pathway Interaction Database [87]), most focus on pathways that are gene-specific (e.g. the NFκB signaling pathway from BioCarta or Cell Signaling Technology [6,16–18]) rather than disease- or tissue-specific [such as the prostate cancer pathway from KEGG (hsa05215) [88–91]]. The construction of novel pathways from a set of genes or the inclusion of novel genes within existing pathways is often based on literature curation [86,87], predictive computational models [92–94], or lab experiments [6,7,18]. In contrast, the association of promising candidate genes with diseases has been widely studied in mutation analyses [95,96] and genome-wide-association studies [97,98], but also in predictive models for disease gene prioritization [99–101] and tissue types [102,103]. Relatively few studies have predicted novel pathways or networks for specific diseases or tissues [104,105], with a more common trend being reporting disease-specific dysregulation in specific pathways of interest [106–109]. Here, we address this challenge and associate NFκB, which is known to be involved in prostate cancer [7] and other diseases [110–112], with a novel predicted pathway activated during prostate cancer.

Although our validation recovered genes known to be associated with NFκB and prostate cancer (S8–S11 Tables) and the majority of our predictions were co-expressed in lethal prostate cancer (S6 and S7 Figs, S15 Table), we did not recover all known genes, e.g. WNT16 or TP53. The WNT16 gene is known to be regulated by NFκB after DNA damage and subsequently activates the canonical Wnt program in prostate tumor cells [113]. The tumor suppressor gene TP53 regulates NFκB in inflammation and cancer more generally [7,114–116]. However, these two genes were not included in our novel NFκB in prostate cancer pathway because their predicted associations with the pathway were not sufficiently confident (below our predefined threshold of 0.9). The low-confidence scores between WNT16, TP53 and NFκB were based on the integrated datasets, as they showed a lower co-expression in the integrated gene expression datasets after regularization in the Bayesian model than the high-confidence genes from the predicted pathway. Therefore, the quality of our predictions depends mainly on the underlying data (e.g. integrated data, gold standard, context-specific gene sets), which is highly influenced by the disease and tissue of interest. While there will be a large amount of disease- and tissue-specific data available for well-studied diseases (e.g. prostate and breast cancer), there is often less data accessible for diseases in which the relevant tissue is difficult to access (e.g. brain- and neurodevelopmental disorders), diseases which are less intensively studied (e.g. rare monogenic diseases), or complex diseases that involve multiple tissues or phenotypes (e.g. diabetes and autism).

Having defined a novel set of biomolecular activities representative of NFκB activation in prostate cancer, two logical next steps would be to 1) experimentally validate their molecular mechanisms of action and 2) evaluate gene sets derived from our extended pathway as potential clinical biomarkers for prostate cancer risk (e.g. in conjunction with criteria such as Gleason 6, low volume PSA < 10). The bulk of newly predicted pathway components represent physical protein-protein interactions: stable co-complexing or transient interactions such as post-translational modifications (e.g. phosphorylation). These should be assayed by extending our preliminary co-immunoprecipitations with additional targets and antibodies, using complex-targeted techniques such as TAP-tagging, and (when possible) specifically assessing protein state by phosphoantibody or mass spectrometry targeting. Transcriptional regulatory predictions, especially those downstream of NFκB itself (e.g. BIRC3, TICAM1), can be more easily assessed by qPCR readout in the presence of knockdown or other perturbations. Each of these targeted experimental readouts could then be re-incorporated into a refined prediction model to further extend or increase confidence in NFκB pathway components.

More importantly, molecular epidemiological data are needed to link these genes’ activities (post-transcriptionally or post-translationally) to prostate cancer severity and outcome. Many patients are not destined to progress to higher grade and potentially lethal disease, and they can thus avoid surgery or radiotherapy if their low risk is detected early by molecular or other biomarkers. Purely expression-based biomarkers of low prostate cancer progression risk have yet to be identified; a more detailed mechanistic perspective (as provided by our predictions) may lead to targeted transcript assays or to sets of informative gene products (e.g. phosphoproteins). Additionally, more nuanced molecular predictors might identify patients with high risk of micrometastatic disease at time of surgery or radiotherapy, who would then be in need of systemic adjuvant therapy to prevent relapse and death. Data assessing the transcriptional and post-translational states of genes in the extended NFκB pathway along with clinical outcome should therefore be collected. The tightly linked pathway components predicted here thus represent one new step along the route to more effective molecular therapies and diagnostics in prostate cancer.

## Methods

To recover the mechanistic pathway components specific to the NFκB pathway as perturbed in prostate cancer (Fig 1), we first integrated high-throughput experimental data from heterogeneous databases and trained our model for specific biological contexts and specific to the NFκB pathway in prostate cancer using NFκB specific pathways as a gold standard and biological contexts which were specific for prostate cancer. Second, we identified genes that were 1) related to NFκB in multiple contexts and with high confidence and 2) differentially expressed in lethal prostate cancer based on an in-house gene expression dataset (Table 1) [25,26]. Third, we trained biomolecular mechanisms for 7 interaction types as defined in an ontology (Fig 4A), which ensured to keep conserved and non-conserved gene pairs in child-parent relationships in the ontology. This step predicted a high-confidence biomolecular mechanism for each functionally related gene pair in the network. Finally, we combined known NFκB-specific genes with the 8 genes differentially expressed in multiple NFκB-related contexts to generate a novel NFκB pathway specific to prostate cancer (Fig 2). All analysis source code and data are available at http://huttenhower.sph.harvard.edu/cap and http://dx.doi.org/10.7910/DVN/WPRDBZ, and for more details on the methods please refer to the S1 Text.

### Integration method

We integrated high-throughput and heterogeneous functional genomic data (see below) using a naïve Bayesian approach with regularization [70,71]. Briefly, as implemented in the Sleipnir library, the process first performs a maximum likelihood count to reconstruct the joint probability distribution for each dataset between its discretized data values and the gold standard of known present and absent functional relationships. Regularization was performed by mixing this joint distribution with a uniform distribution using weights proportional to the normalized mutual information shared between the dataset and all other datasets to be integrated; for details, see [70]. This parameter regularization ensures that datasets that contain unique information are upweighted, while datasets that contain common information are downweighted to prevent “overconfidence” due to the naïve Bayes independence assumption. We trained one classifier for each biological context and each interaction mechanism individually, using the corresponding gold standard (see below) as the underlying ground truth in the training and testing process.

#### Context-specificity

To predict context-specific functional networks for a set of 9 relevant biological contexts (S3 Table), we first specified an individual gold standard for each context (see below). Context-specificity was defined as in [70] by drawing gene sets from each of nine Gene Ontology [117] terms: cell death, regulation of cell proliferation, regulation of cell differentiation, cell migration, regulation of cell cycle, vascular development, regulation of cell motion, cytokine metabolic process, and positive regulation of NFκB. We produced once predicted functional relationship network for each context-specific gold standard. We extracted high-confidence subgraphs around the NFκB1 gene in each of these networks, resulting in nine context-specific subgraphs consisting of genes highly functionally related to NFκB1 (see, for example, Fig 3A). Subgraph queries were performed as in [70] by identifying the 40 network neighbors connected with greatest specificity (highest ratio of intra- to inter-group edge weight) to the original query genes. Here, we used the NFκB1 gene as the sole query gene. This identified 66 genes functionally related to NFκB1 in at least two different biological contexts (Fig 3A, S5 Table), of which eight were significantly down-regulated in prostate cancer microarray experiments (see below, Table 1).

#### Mechanism-specificity

To assign a biomolecular mechanism to each functionally related gene pair in the final network, we constructed an interaction ontology consisting of seven interaction types and applied an integrated method for concurrently predicting multiple protein interaction types [24] (Fig 4A). Within our defined interaction ontology a functional relationship can be a physical interaction (two genes directly bind to each other and interact physically), a complex (two genes form a protein-binding complex), a covalent modification (a kinase posttranslationally modifies a substrate), a phosphorylation (a kinase adds a phosphate group to a substrate at a phosphorylation site), a shared pathway (two genes react in the same pathway which can be an indirect regulation), a regulatory interaction (a gene is activating or inhibiting another gene), or a synthetic interaction (two genes simultaneously regulate another gene, whereas the two genes individually would not regulate the third gene).

Next, we used an integrated method for concurrently predicting multiple protein interaction types [24] to assign a biomolecular mechanism to each functionally related gene pair in the final network. Based on a multi-label hierarchical classification formulation [24,75], we learned an individual Bayesian classifier for each interaction type (see above) using the corresponding mechanistic gold standard for the interaction type for training (see below, S4 Table). After training these 7 individual classifiers, we constructed a Bayesian network based on the ontology structure and fixed conditional parameters to constrain the hierarchical semantics of the ontology [24]. This algorithm ensures to keep conserved and non-conserved gene pairs in child-parent relationships in the ontology. This step revealed a high-confidence biomolecular mechanism for each functionally related gene pair in the network.

Finally, we generated the novel NFκB pathway by extracting high-confidence subnetworks from each individual interaction network using 18 query genes—including the NFκB complex genes (NFκB1, NFκB2, REL, RELA, and RELB), their inhibitors (IκB-α/ε, IKK-α/β/γ), and eight genes significantly down-regulated in lethal prostate cancer (see above, Tables 1 and S12)—and a neighborhood query size of *k = 10* using the HEFalMp ratio query algorithm (see above). Next, we integrated these subgraphs into one pathway in which genes were connected by high-confidence biomolecular mechanisms (Fig 2). This integration of 7 mechanism-specific subgraphs resulted in a single pathway containing 50 genes, which were connected by 112 non-redundant biomolecular mechanisms (as defined in Fig 4A and illustrated in Fig 2).

### Integrated data

We incorporated 633 baseline microarray expression datasets from the NCBI Gene Expression Omnibus repository (GEO) [118] as identified in [70]. These comprised 14,617 individual conditions, to which we further added 18 human gene expression datasets identified by ARepA [119] as containing the phrase “prostate tumor” or “prostate cancer” in their metadata annotations (S1 Table). All data acquisition, processing, and normalization were performed using ARepA’s default parameters, specifically 1) RMA normalization using the R/affy package [120], 2) co-expression using *z*-score normalized Pearson correlation [70,71], and 3) gene identifier harmonization using BridgeDB [121].

We computed a normalized correlation measure for each gene pair in each dataset to assess a similarity score as co-expression for all gene pairs [70,71,119]. In addition to gene expression assays, we collected 225 non-microarray datasets from the protein interaction databases BioGRID [122], IntAct [123], STRING [124], Prosite [125], Domine [126], Transfac [127], and ORegAnno [128], which collectively contained 1,351,782 pairwise gene interactions (S6 Table) derived from 878 datasets.

### Gold standard

To generate a gold standard specific to the NFκB pathway, we manually chose 30 pathways from the PathwayCommons database [129] (S2 Table) that 1) contained the NFκB1 gene, 2) were non-redundant, and 3) contained at most 200 genes. This collection of known NFκB1 pathways was converted into a set of 57,533 related (positive or related) gene pairs, to which the same quantity of random (negative or not related) gene pairs was added to generate both positive and negative gold standards for use in the data integration process described below.

#### Context-specific gold standard

Out of 442 biological processes from Gene Ontology, our medical team manually chose nine biological contexts that play an important role in human prostate cancer development and progression: cell death, cell differentiation, cell cycle, cell proliferation, cell migration, cell motion, vascular development, cytokine metabolic process, or NFκB regulation. These contexts consist of gene lists with a number of genes ranging from 37 to >1,000 (S3 Table). For each of these contexts we derived a set of related genes based on GO annotations (S3 Table). To associate these context-specific gene sets with prostate cancer, we downloaded 12,544 significantly up- and down-regulated genes (FDR < 0.05) in human prostate carcinoma from the Gene Expression Atlas (a resource that meta-analyzes a curated subset of microarray expression datasets from the ArrayExpress database for condition-specific gene expression patterns [73]). Next, we used this set of genes to refine our niner biological contexts into contexts specific to prostate cancer by considering only up- or down-regulated genes within the contexts (S3 Table). To finally generate context-specific gold standards specific to prostate cancer, we refined our global gold standard dataset by decomposing it into subsets related to each of these contexts specific to prostate cancer, resulting in nine gold standards specific to relevant biological context and the NFκB pathway in prostate cancer.

#### Mechanism-specific gold standard

As our interaction ontology consists of seven hierarchically organized biological mechanisms (Fig 4A), we accordingly defined interaction mechanism-specific gold standards. These mechanism-specific related (positive) gene pairs were retrieved from the PathwayCommons database [129], the Human Protein Reference Database (HPRD) [130], Transfac [127] and ORegAnno [128] (S4 Table). Additionally, due to the interaction hierarchy, interaction parents (e.g. physical interaction) inherited known positive interactions from their interaction children (e.g. covalent modification and complex), with equal amounts of random (negative or not related) gene pairs added to represent negative (non-occurring) interactions. This resulted in 34,796 positively related interacting gene, with mechanismspecific gold standard sizes ranging from 722 interactions (synthetic interaction) to 24,034 interactions (shared pathway interaction; S4 Table).

### Gene expression profiling

#### Gene expression profile in lethal prostate cancer

We used a gene expression dataset which is based on a set of four complementary DNA (cDNA)–mediated annealing, selection, ligation, and extension (DASL) assay panels (DAPs) for the discovery of molecular signatures relevant to prostate cancer for 116 male patients with prostate cancer from an inhouse Physicians^’^ Health Study (PHS) Prostatectomy Confirmation Cohort from the United States [25,26]. To assess the differential expression between lethal and indolent subgroups, we computed fold changes and corresponding *p*-values using the R/limma package, revealing 186 out of 6,096 genes as differentially expressed at a significance level of 5% after FDR correction (S7 Table).

#### TCGA gene expression dataset for co-expression in prostate cancer

We obtained RNA-Seq Level 3 data from the October 12^th^, 2013 Broad Institute Firehose run (http://gdac.broadinstitute.org). We applied RSEM abundance quantification at the gene level, where values were normalized to set the upper quartile count at 1,000 reads. A complete description of the TCGA data processing pipeline is available at the TCGA data portal (https://tcga-data.nci.nih.gov) in the MAGE-TAB annotation files. Next, this dataset was integrated into a single prostate cancer-specific co-expression network using unsupervised data integration averaging across normalized co-expression values (*z*-scores) [71].

### Computational evaluation

#### Estimating true and false positive predictions in a quality control analysis

We computationally estimated the true positive rate and precision of our predictions by comparing with known interaction mechanisms from 1) the NFκB signaling pathway in BioCarta (BioCarta.com, BioCarta LLC) and literature, 2) NCBI, and 3) prostate cancer-specific co-expression analysis based on the TCGA dataset (see above).

First, we compared predictions from our Bayesian network model based on the interaction ontology (Fig 4A) with 31 known biomolecular mechanisms extracted from the NFκB signaling pathway in BioCarta and literature (S8 and S9 Tables). For each gene pair with a known mechanism from BioCarta we extracted corresponding predicted mechanism(s) from our mechanism-specific networks using a threshold of 0.96 (this value represents the top 5% most confident predicted gene interactions; S8 Table). By comparing the known mechanisms with their corresponding predictions, we estimated the number of true positives (TP), which are predictions that match the known mechanisms, and false positives (FP), which are predictions that are not among the known mechanisms. The estimation of the number of true negatives (TN) based on the non-existing edges in the NFκB signaling pathway in BioCarta, resulted in a set of 28 gene interactions which are not existing according to BioCarta (S9 Table). This comparison resulted in 23 TP out of 31 known associations (TPR = 74%) and a precision rate of 33%. However, since the information that we extracted from BioCarta is incomplete, we extended our list of known interactions by mechanisms that have been mentioned in the literature. In doing so, we observed a higher TPR and precision (81% and 67%, respectively). This improvement was due to an increase in the number of known (positive) interaction mechanisms when incorporating literature support, many of which were also predicted with high confidence by our model.

Second, we compared 50 known mechanisms from the literature with our predictions. Here, we chose the top 50 mechanisms (25 for each NFκB1 and TNF) which were absent from the gold standard and for which literature evidence was available from NCBI gene catalogue (S10 and S11 Tables). As before, we extracted all high-confidence predicted interaction mechanisms for these 50 gene pairs at a threshold of 0.96.

Third, we compared our high-confidence predicted interactions from the novel NFκB pathway with co-expression data derived from TCGA. Using these data, we computed co-expression values for all gene pairs and compared the derived *p*-values with our confidence scores for all 112 predicted interactions in the pathway. As a negative control, we randomly selected the same number of predicted interactions with low predicted confidence scores from the genome.

### Interaction validation by co-immunoprecipitation and knockdown

We performed two screens for NEDD9 activity and ZFP36 interaction: co-immunoprecipitation and siRNA knockdown in a prostate cancer cell line. LAPC4 cells were received from Dr. Robert Reiter, University of California, Los Angeles. These were maintained in RPMI 1640 at 37°C, 5% CO2, and 100% relative humidity and supplemented with 10% FBS and 100 IU of penicillin and streptomycin (100 μg/ml).

For NEDD9 / ZFP36 interaction testing, LAPC4 cells were lysed by RIPA buffer and protein concentration was measured by protein BCA assay (Bio-Rad). Cell lysis of 500 μg was applied for each immuno-precipitation for NEDD9 and ZFP36. Rabbit IgG was included as a control and the results were analyzed by western blot with NEDD9 (Fisher Scientific).

For knockdown, we read out cell proliferation as a phenotype using LAPC4 cells cultured until ~80% confluence and then transfected with siRNAs (Origene) using lipofectamine 2000. NEDD9 siRNA probes 1 through 3 were purchased as catalog #SR303132 with sequences CCCAAGAACAAGAGGUAUAUCAGGT, GGCCUUAUAUGACAAUGUCCCAGAG, and CAACAGAAGCUCUAUCAAGUGCCAA, respectively. Knockdown efficiency was detected by western blot at 3 days after transfection. For cell proliferation, cells were split into 96-well plate with a confluence of ~40% after siRNA transfection for 24 hours. The cell proliferation assay was carried out at different days after splitting using the WST-1 assay (Roche) with the detection of the absorption at a wavelength of 450 nm (following manufacture instructions). Each experiment was performed in triplicate.

## Supporting Information

S1 Fig Scale-free distribution in all 8 mechanistic interactomes was revealed by plotting the node degree against the node density on a log-log scale. (PDF)

S2 Fig Analysis of the extent of the overlap (Jaccard Index) of the top 5% of high-degree hubs between all 8 mechanistic interactomes show that hub genes differ across biomolecular mechanisms but show high similarity among related mechanisms. (PDF)

S3 Fig The performance of the context-specific networks (AUC values) does not depend on the number of genes in the context-specific gene sets for refining the gold standard necessary for training the context-specific functional relationship networks. (PDF)

S4 Fig The performance of the interaction networks (AUC values) does not depend on the number of genes in the interaction specific gene sets for refining the gold standard necessary for training the interaction networks. (PDF)

S5 Fig To estimate the number of true positive (solid lines) and false positive (dotted lines) predictions, we compared our predictions with 31 known biomolecular mechanisms extracted from the NFκB signaling pathway in BioCarta (BioCarta.com, BioCarta LLC). For each gene pair with a known interaction mechanism we extracted their corresponding predicted mechanism(s) from our predicted interaction networks using a threshold of 0.96 (representing the top 5% highest-confidence predictedgene interactions).
(PDF)

S6 Fig The performance of the predictions from the newly predicted NFκB pathway in comparison with co-expression as retrieved from the TCGA dataset (see Methods). (PDF)

S7 Fig The performance of the predictions from the newly predicted NFκB pathway in comparison with co-expression as retrieved from the TCGA dataset (see Methods). Top: Comparison of all high-confident predictions from the novel NFκB pathway with co-expression values as retrieved from the TCGA dataset, revealing 70% of predicted high-confidence interactions in this pathway to be co-expressed in the TCGA dataset as well (see S15 Table). Bottom: Comparison of random negative predictions from the genome (1:1 positive:negative ratio) with co-expression values as retrieved from the TCGA dataset, revealing that <10% randomly chosen low-confident predictions were not significantly co-expressed in the TCGA dataset (see S15 Table).
(PDF)

S1 Table A list of all 18 manually curated microarray expression data sets chosen to be particularly informative for functional relationships in prostate cancer. (XLSX)

S2 Table A list of 30 manually chosen non-redundant pathways from the PathwayCommons database [129] used to generate a gold standard specific to the NFκB pathway. All contain the NFκB1 gene and at most 200 genes.
(XLSX)

S3 Table A list of 9 manually chosen biological contexts (out of 442 biological processes from Gene Ontology) that play an important role in human prostate cancer development and progression, including cell death, cell differentiation, cell cycle, cell proliferation, cell migration, and NFκB regulation. To make the contexts prostate cancer-specific, we overlapped the corresponding gene sets with differentially expressed genes from the GXA database (see Methods).
(XLSX)

S4 Table For each of the seven hierarchically organized biological mechanisms from the interaction ontology (Fig 4A), we accordingly defined mechanism-specific gold standards from the PathwayCommons database [129], the Human Protein Reference Database (HPRD) [130], Transfac [127] and ORegAnno [128]. Due to the interaction hierarchy, interaction parents (e.g. physical interaction) inherited known positive interactions from their interaction children (e.g. covalent modification and complex), with equal amounts of random (negative or not related) gene pairs representing negative interactions.
(XLSX)

S5 Table A list of 66 genes that predicted to be highly functionally related with NFκB1 in multiple biological processes (S3 Table). (XLSX)

S6 Table In addition to 633 non-disease and 18 human prostate cancer-specific microarray expression datasets from GEO, we further collected 225 non-microarray datasets from the protein interaction databases BioGRID [122], IntAct [123], STRING [124], Prosite [125], Domine [126], Transfac [127], and ORegAnno [128], together containing 1,351,782 pairwise gene interactions. (XLSX)

S7 Table 186 out of 6,096 genes were significantly differently expressed at a significance level of 5% after FDR correction in our underlying Physicians^’^ Health Study (PHS) Prostatectomy Confirmation Cohort [25,26]. (XLSX)

S8 Table To estimate the true positive rate and precision of our predictions, we compared with 31 known biomolecular mechanisms extracted from the NFκB signaling pathway in BioCarta (BioCarta.com, BioCarta LLC). For each gene pair with a known mechanism from BioCarta we extracted their corresponding predicted mechanism(s) from our corresponding predicted interaction networks (using a threshold at 0.96 as this value representing the top 5% highest-confidence gene interactions). By comparing known mechanisms with their corresponding predictions, we estimated the number of true positives (TP), which are predictions that match the known mechanisms, and false positives (FP), which are predictions that are not among the known mechanisms.
(XLSX)

S9 Table To estimate the number of true negatives (TN), we compared our predictions with 28 non-existing edges in the NFκB signaling pathway in BioCarta. (XLSX)

S10 Table To estimate the true positive rate and precision of our predictions, we compared 25 known biomolecular mechanisms for the NFκB1 gene as extracted from the NCBI gene catalogue (http://www.ncbi.nlm.nih.gov/gene?db=gene&report=generif&term=4790 accessed 9/20/2013) which were also absent from the gold standard. We again extracted all high-confidencemechanisms for all gene pairs at a threshold level of 0.96 representing the top 5% highest-confidence gene interactions.
(XLSX)

S11 Table To estimate the true positive rate and precision of our predictions, we compared 25 known biomolecular mechanisms for the TNF gene as extracted from the NCBI gene catalogue (http://www.ncbi.nlm.nih.gov/gene?db=gene&report=generif&term=7124 accessed 09/20/2013) which were also absent from the gold standard. We again extracted all high-confidencemechanisms for all gene pairs at a threshold level of 0.96 representing the top 5% highest-confidence gene interactions.
(XLSX)

S12 Table For generating our novel NFκB pathway, we used a set of 18 query genes, including five NFκB complex genes, five NFκB inhibitor genes, and eight differentially expressed and highly NFκB-related genes in mulitple cancer-related contexts (Table 1). (XLSX)

S13 Table We analyzed the 40 newly predicted genes (excluding 5 NFκB complex genes and 5 NFκB inhibitor genes, see S12 Table) (A) from our novel NFκB pathway for gene set enrichments [27] on Gene Ontology categories, pathways from KEGG and NCI, and diseases from the NIH Cancer Gene Index (B). (XLSX)

S14 Table Performance of context (A) and interaction (B) networks based on a 10-fold cross validation. The AUC values are the median values across all 10 cross validations per mechanism.
(XLSX)

S15 Table We compared our predictions with co-expression relationships inferred from the TCGA dataset (see Methods). (A) Comparison of all high-confidence predictions from the novel NFκB pathway (Fig 2) with co-expression values as retrieved from the TCGA dataset, revealing 70% of all predicted high-confidence interactions in this pathway to be co-expressed in the TCGA dataset as well. (B) Comparison of random negative predictions from the genome (1:1 positive:negative ratio) with co-expression values as retrieved from the TCGA dataset, revealing that <10% randomly chosen low-confident predictions were significantly co-expressed in the TCGA dataset. For visualization see S6 and S7 Figs.
(XLSX)

S16 Table List of all genes from our novel predicted NFκB pathway and their gene annotations, including 1) gene description (red color indicates promising and novel candidate gene, blue color indicates genes that are known to be associated with NFκB or lethal prostate cancer that we recovered in the pathway); 2) differential expression as described in the Methods section [25,26] (red color indicates significantly differentially expressed at a significant level of 5%); 3) information about our predictions, such as suggested mechanisms with NFκB within the pathway, whether the gene is predicted to act as a downstream or upstream regulator, activator or suppressor, or oncogene or tumor suppressor gene; 4) gene expression (significant up- or downregulation) from GXA for normal prostate tissue, prostate cancer and immune system ("X" indicates significantly up- or downregulated in GXA);5) genes that are known to be NFκB pathway members [7];6) information about being known NFκB target gene or regulator from 2 different sources (Broad Institute, [131]); 7) known relationship with NFκB and/or prostate cancer; and 8) pathways from KEGG, BioCarta, NCI, and Reactome in which the genes are known to act. (XLSX)

S17 Table Comparison of predicted mechanisms in the NFκB pathway (Fig 2) with existing knowledge based on (A) gold standard and (B) literature comparison. For some predicted interactions, there is no such interaction known, but there is evidence that both interactors are upregulated together (C). Here, out of 112 predicted mechanisms, we observed 13 (11%) to be present in the gold standard, while 29 (26%) were confirmed in the literature, and 18 (16%) were reported to be coregulated.
(XLSX)

S1 Text A detailed description of the methodology. (PDF)
