# Supplementary material for: Computational Reconstruction of NFκB Pathway Interaction Mechanisms during Prostate Cancer
Source: PLoS Comput Biol. 2016 Apr 14;12(4):e1004820. doi: 10.1371/journal.pcbi.1004820 (PMC4831844; doi:10.1371/journal.pcbi.1004820)
Supplement: S1 Fig — (PDF) [file pcbi.1004820.s001.pdf]

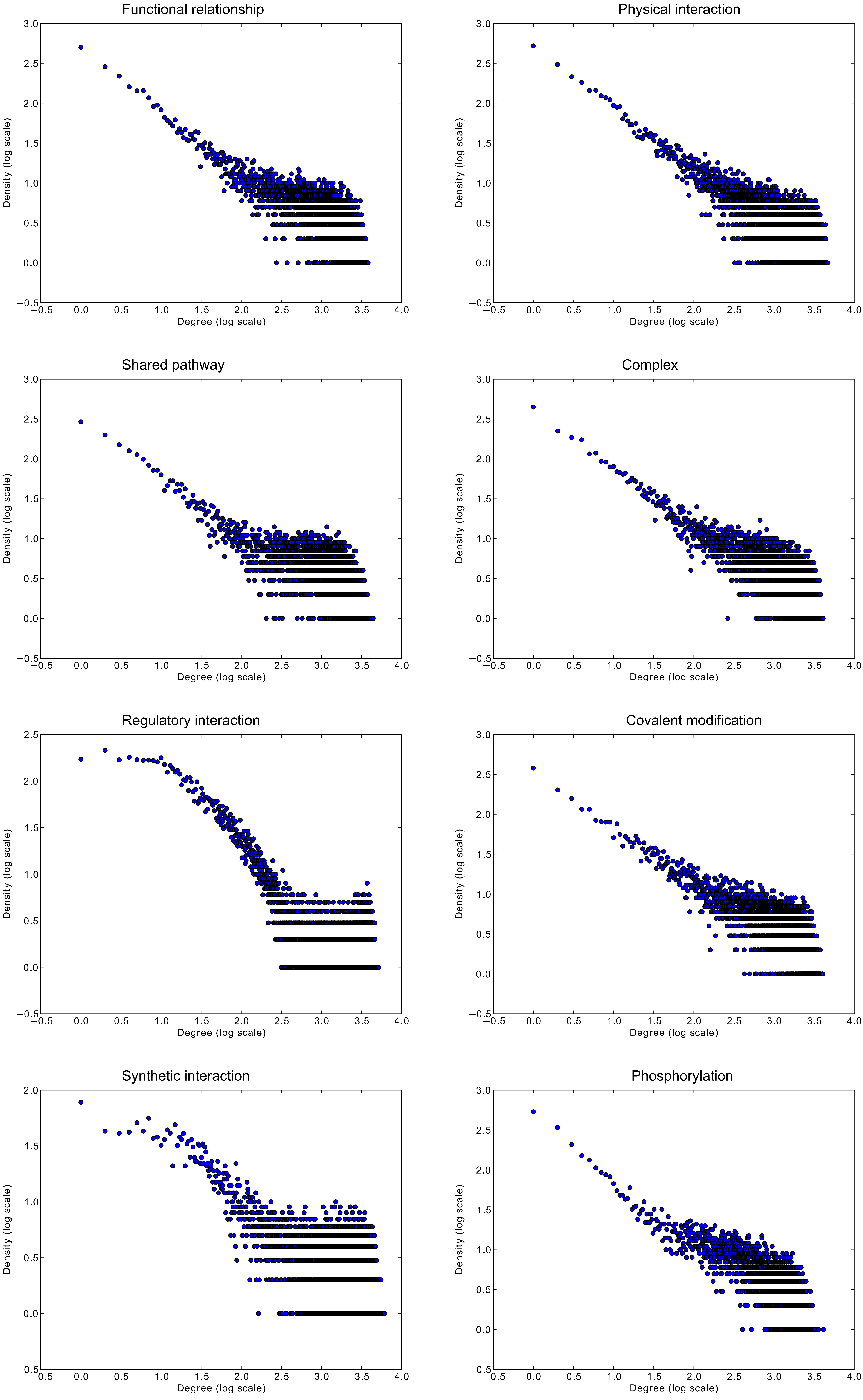
Functional relationship
Physical interaction
Shared pathway
Complex
Regulatory interaction
Covalent modification
Synthetic interaction
Phosphorylation
Density (log scale)
Degree (log scale)
