# Supplementary figures and images for: Computational Reconstruction of NFκB Pathway Interaction Mechanisms during Prostate Cancer
Source: PLoS Comput Biol. 2016 Apr 14;12(4):e1004820. doi: 10.1371/journal.pcbi.1004820 (PMC4831844; doi:10.1371/journal.pcbi.1004820)

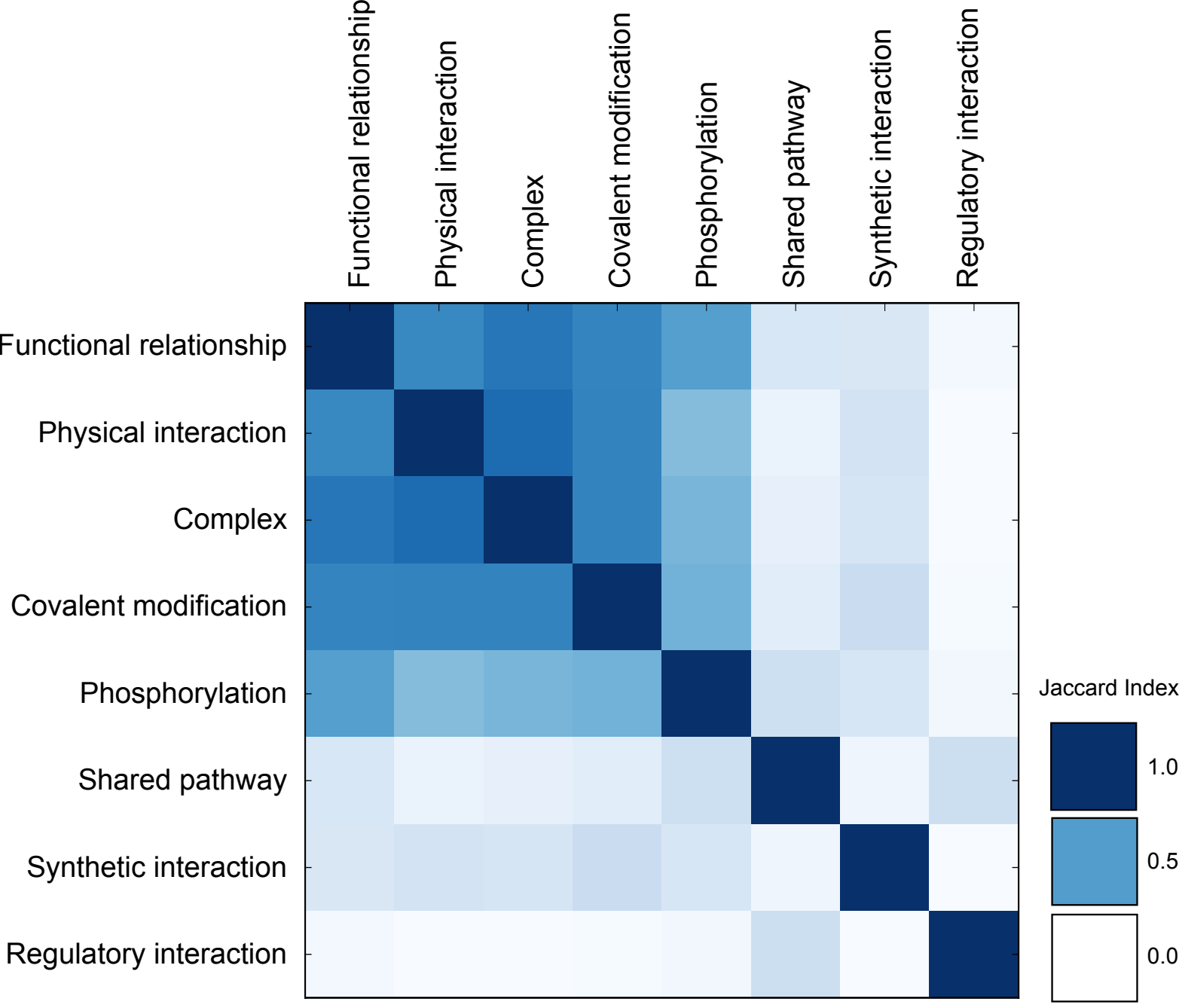

Supplement: S2 Fig — (PDF) [file pcbi.1004820.s002.pdf]

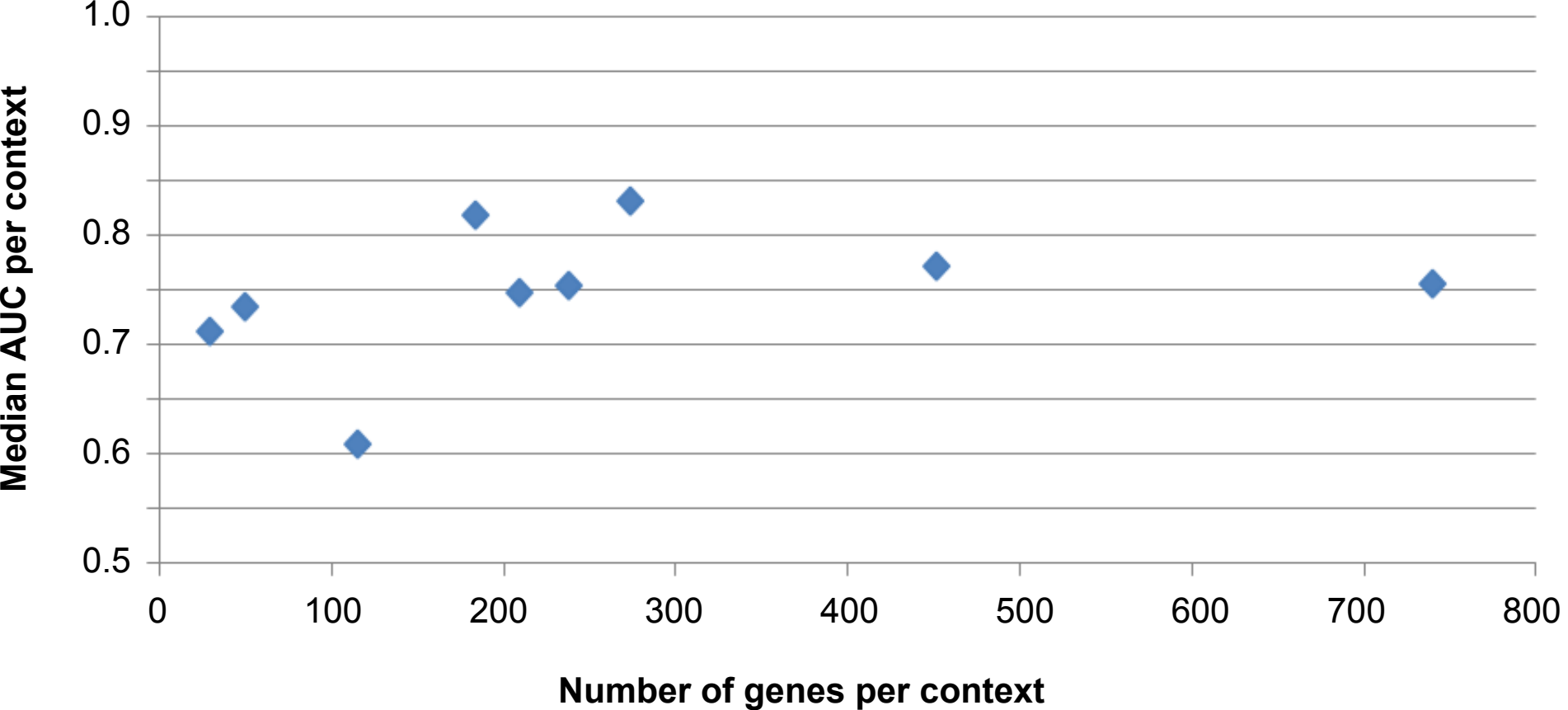

Supplement: S3 Fig — (PDF) [file pcbi.1004820.s003.pdf]

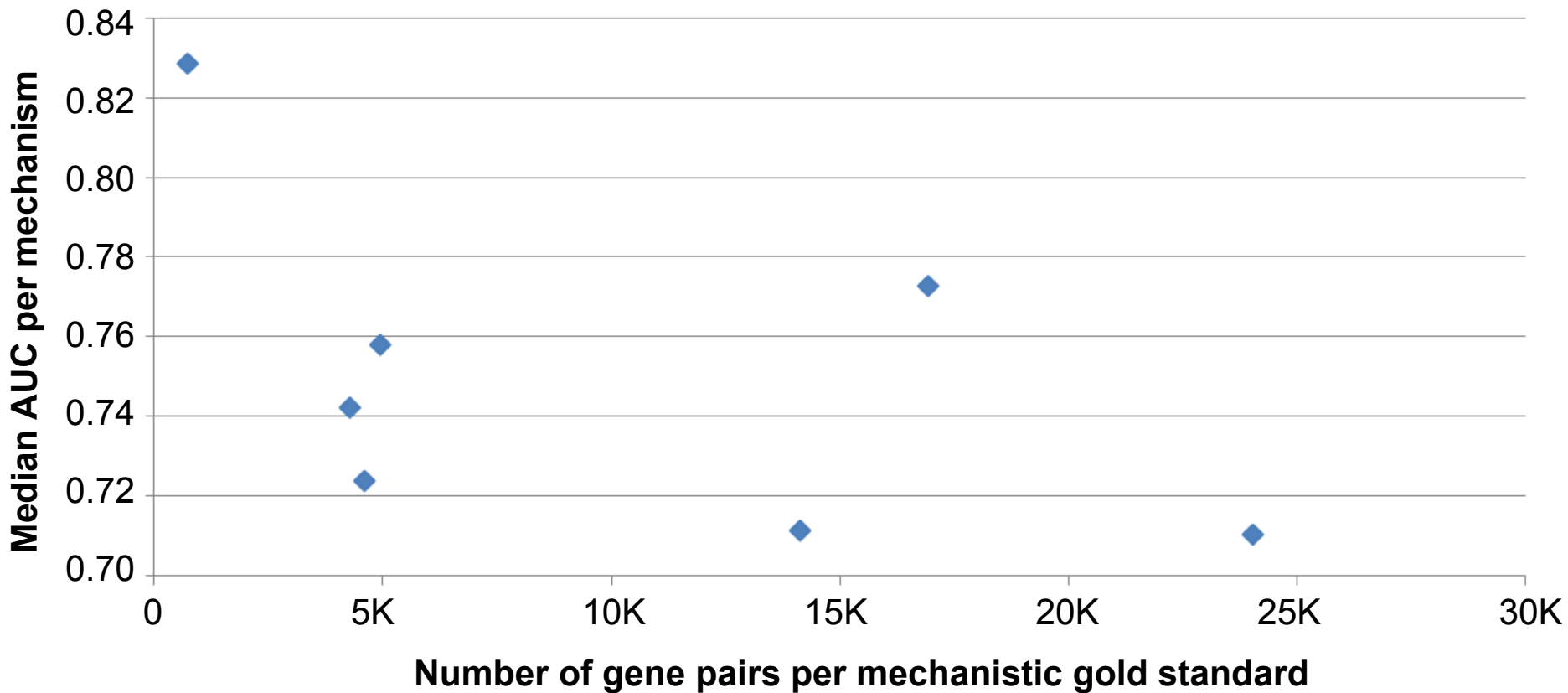

Supplement: S4 Fig — (PDF) [file pcbi.1004820.s004.pdf]

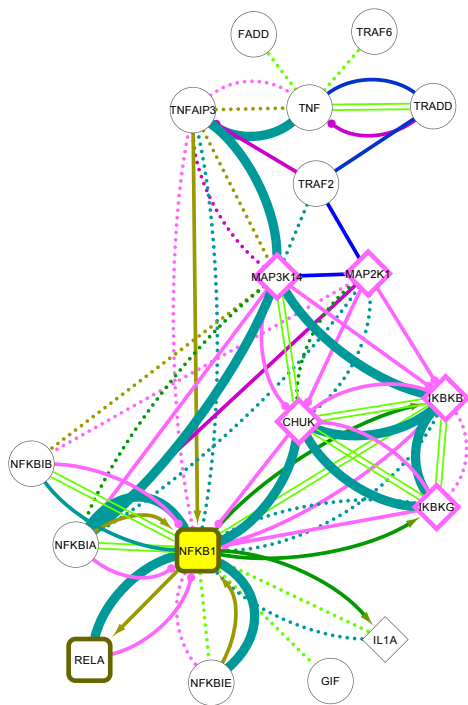

True positive  
predictions

False positive  
predictions

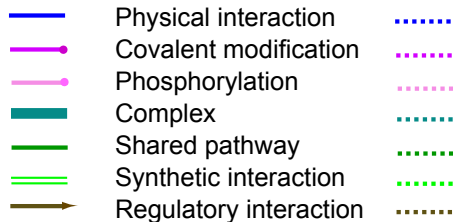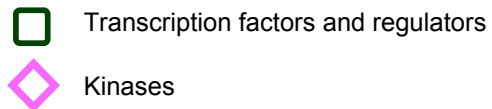

Supplement: S5 Fig — For each gene pair with a known interaction mechanism we extracted their corresponding predicted mechanism(s) from our predicted interaction networks using a threshold of 0.96 (representing the top 5% highest-confidence predictedgene interactions). (PDF) [file pcbi.1004820.s005.pdf]

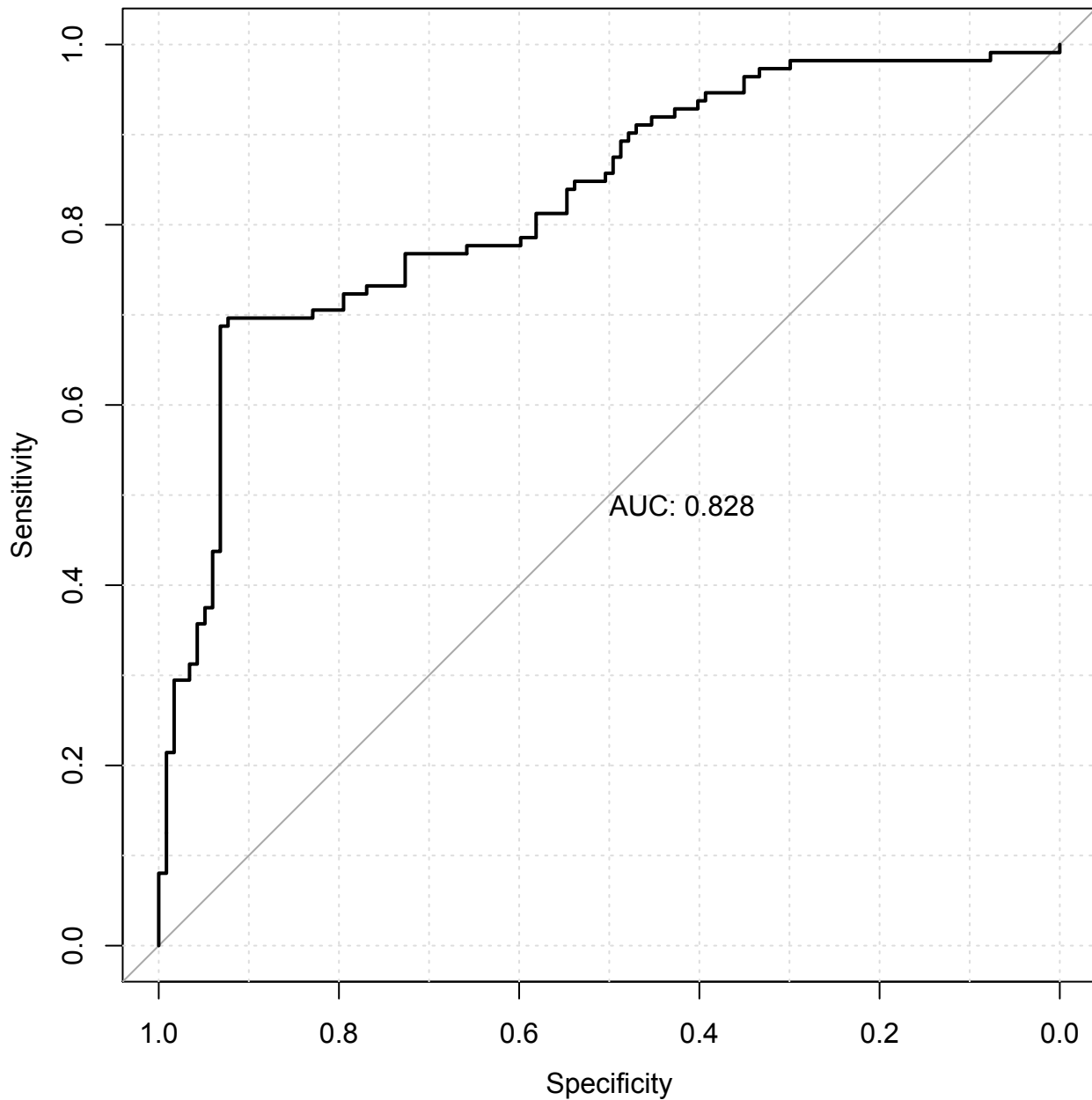

Supplement: S6 Fig — (PDF) [file pcbi.1004820.s006.pdf]
