# Supplementary material for: Computational Reconstruction of NFκB Pathway Interaction Mechanisms during Prostate Cancer
Source: PLoS Comput Biol. 2016 Apr 14;12(4):e1004820. doi: 10.1371/journal.pcbi.1004820 (PMC4831844; doi:10.1371/journal.pcbi.1004820)
Supplement: S7 Fig — Top: Comparison of all high-confident predictions from the novel NFκB pathway with co-expression values as retrieved from the TCGA dataset, revealing 70% of predicted high-confidence interactions in this pathway to be co-expressed in the TCGA dataset as well (see S15 Table). Bottom: Comparison of random negative predictions from the genome (1:1 positive:negative ratio) with co-expression values as retrieved from the TCGA dataset, revealing that <10% randomly chosen low-confident predictions were not significantly co-expressed in the TCGA dataset (see S15 Table). (PDF) [file pcbi.1004820.s007.pdf]

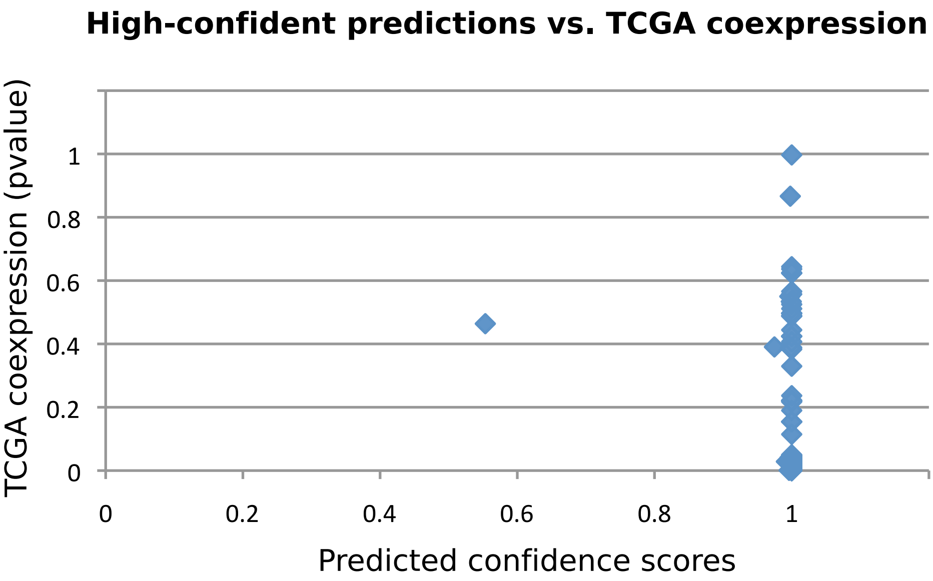
High-confident predictions vs. TCGA coexpression
TCGA coexpression (pvalue)
Predicted confidence scores
0
0.2
0.4
0.6
0.8
1

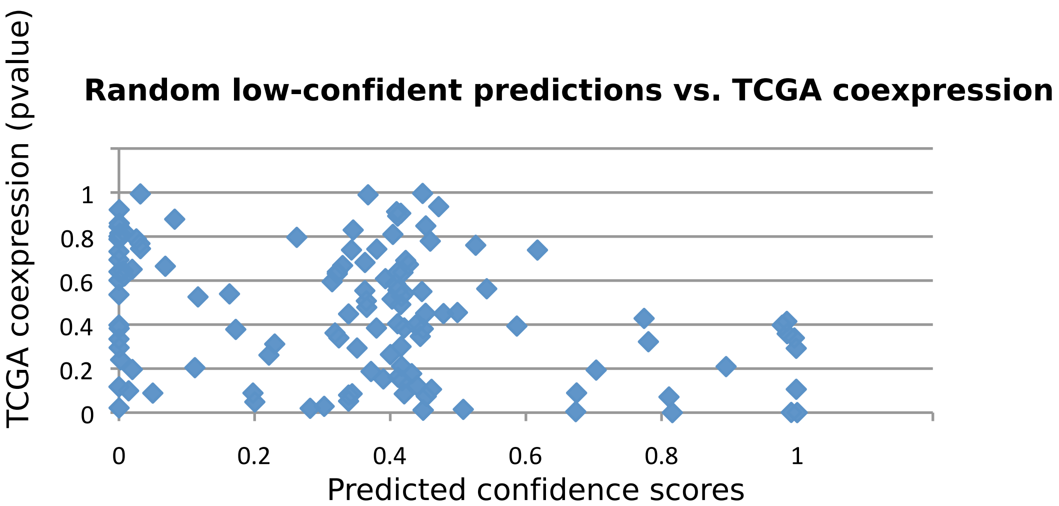
Random low-confident predictions vs. TCGA coexpression
TCGA coexpression (pvalue)
Predicted confidence scores
0
0.2
0.4
0.6
0.8
1
