# Supplementary material for: Computational Reconstruction of NFκB Pathway Interaction Mechanisms during Prostate Cancer
Source: PLoS Comput Biol. 2016 Apr 14;12(4):e1004820. doi: 10.1371/journal.pcbi.1004820 (PMC4831844; doi:10.1371/journal.pcbi.1004820)
Supplement: S1 Text — (PDF) [file pcbi.1004820.s025.pdf]

Supplementary Text

Börnigen et al. (2015)

"Computational reconstruction of NFkB pathway interaction mechanisms during prostate cancer"

1. **Data collection**
   a. **Heterogeneous data**
   - We incorporated 633 baseline microarray expression datasets from the NCBI Gene Expression Omnibus repository (GEO): http://huttenhower.sph.harvard.edu/sites/default/files/public/GEO_datalist.txt.
   - We collected 18 human gene expression datasets specific to prostate cancer: http://huttenhower.sph.harvard.edu/sites/default/files/public/GEO_cap_datalist.txt or Supplementary Table 1.
   - We collected 225 nonmicroarray datasets from the protein interaction databases BioGRID, IntAct, STRING, Prosite, Domine, Transfac, and ORegAnno (Supplementary Table 6).

   All input datasets were downloaded, processed, normalized and standardized using ARepA (http://huttenhower.sph.harvard.edu/arepa). For all gene expression datasets, we computed a normalized correlation measure for each gene pair in each dataset to assess a similarity score as co-expression for all gene pairs.

   b. **Gold standard**
   We manually chose 30 pathways from the PathwayCommons database that contain the NFκB1 gene and that are non-redundant with at most 200 genes in each pathway:
   http://huttenhower.sph.harvard.edu/sites/default/files/public/answerer.txt
   or Supplementary Table 2.

   c. **Context-specific gene sets**
   From Gene Ontology we manually chose 9 biological contexts that play an important role in human prostate cancer development and progression:
   http://huttenhower.sph.harvard.edu/sites/default/files/public/contexts.zip
   or Supplementary Table 3.
   These contexts consist of gene lists with a number of genes.

   d. **Mechanistic gene sets**
   For each biological mechanism from our interaction ontology (Figure 4A), we defined mechanism specific gold standards from PathwayCommons, the Human Protein Reference Database (HPRD), Transfac and ORegAnno:

http://huttenhower.sph.harvard.edu/sites/default/files/public/interactionmechanisms.zip or Supplementary Table 4.

e. **Meta-analyzed human prostate cancer gene expression data**

We downloaded 12,544 significantly down- and up-regulated genes (FDR < 0.05) for human prostate carcinoma from the Gene Expression Atlas which meta-analyzes a curated subset of microarray expression datasets from the ArrayExpress database for condition-specific gene expression patterns.

f. **Gene expression profile in lethal prostate cancer**

We used a gene expression dataset for the discovery of molecular signatures relevant to prostate cancer for 116 male patients with prostate cancer from an inhouse Physicians' Health Study (PHS) Prostatectomy Confirmation Cohort from the United States. To assess the differential expression between lethal and indolent subgroups, we computed fold changes and corresponding p-values using the R/limma package (Supplementary Table 7).

2. **Generate context-specific and mechanism-specific gold standards**

a. **Context-specific gold standard**

To associate the context specific gene sets with prostate cancer, we used the set of meta-analyzed human prostate cancer genes to refine our 9 biological contexts into contexts specific to prostate cancer by considering only up- or down-regulated genes within the contexts (Supplementary Table 3). To finally generate context specific gold standards specific to prostate cancer, we refined our global gold standard specific (see above) by decomposing it into subsets related to each of these contexts specific to prostate cancer, resulting in 9 gold standards specific to relevant biological context and the NFκB pathway in prostate cancer.

b. **Mechanism-specific gold standard**

As our interaction ontology consists of 7 hierarchically organized biological mechanisms (Figure 4A), we accordingly defined mechanism specific gold standards (Supplementary Table 4). Additionally, due to the interaction hierarchy, interaction parents (e.g. physical interaction) inherited known positive interactions from their interaction children (e.g. covalent modification and complex), with equal amounts of random (negative or not related) gene pairs representing negative interactions.

3. **Bayesian data integration**

We integrated high-throughput and heterogeneous genomics data using a naïve Bayesian approach with regularization. Briefly, as implemented in the Sleipnir library, the process first performs a maximum likelihood count to reconstruct the joint probability distribution for each dataset between its discretized data values and the gold standard of known present and absent functional relationships. Regularization was performed by mixing this joint distribution with in a uniform distribution using weight proportional to the normalized mutual information shared between the dataset and all other datasets to be integrated. This parameter regularization ensures that datasets that contain informative and unique

information are upweighted, while datasets that contain shared information across many datasets are downweighted to estimate the uniqueness of their contributions and prevent "overconfidence" do to the naïve Bayes independence assumption. We trained one classifier for each biological context and each interaction mechanism individually, using the corresponding gold standard as the underlying ground truth in the training and learning process.

4. **High-confidence NFkB network**

   We extracted high-confidence subgraphs around the NFκB1 gene in each of these context-specific networks, resulting in nine context-specific subgraphs consisting of genes highly functionally related to NFκB1 (see e.g. Figure 3A). Subgraph queries were performed by identifying the 40 network neighbors connected with greatest specificity (highest ratio of intra- to inter-group edge weight) to the original query genes. Here, we used the NFκB1 gene as the sole query gene. This identified 66 genes functionally related to NFκB1 in at least two different biological contexts (Figure 3A, Supplementary Table 5), among which eight genes were significantly down-regulated in prostate cancer microarray experiments (Table 1).

5. **Biomolecular mechanisms**

   We used an integrated method for concurrently predicting multiple protein interaction types to assign a biomolecular mechanism to each functionally related gene pair in the final network. Based on a multi-labeled hierarchical classification formulation, we learned an individual Bayesian classifier for each interaction type (see above) and using the corresponding mechanistic gold standard for the interaction type for training (Supplementary Table 4). After training these 7 individual classifiers, we constructed a Bayesian network based on the ontology structure and fixed conditional parameters to constrain the hierarchical semantics of the ontology. This algorithm ensures to keep conserved and non-conserved gene pairs in child-parent relationships in the ontology. This step revealed a high-confidence biomolecular mechanism for each functionally related gene pair in the network.

6. **NFkB pathway in prostate cancer**

   Finally, we generated the novel NFκB pathway by extracting high-confidence subnetworks from each individual interaction network using 18 query genes, including the NFκB complex genes (NFκB1, NFκB2, REL, RELA, and RELB), their inhibitors (IκB-α/ε, IKK-α/β/γ), and 8 genes significantly down-regulated in lethal prostate cancer (Table 1, Supplementary Table 12) and a neighborhood query size of *k=10* using the HEFalMp ratio query algorithm (see above). Next, we integrated such generated subgraphs into one pathway in which genes were connected by high-confidence biomolecular mechanisms (Figure 2).
